# CD8^+^ T cells in the tumor microenvironment modulate the response to endocrine therapy in breast cancer

**DOI:** 10.1172/JCI188458

**Published:** 2025-12-09

**Authors:** Fabiana Napolitano, Yunguan Wang, Dhivya R. Sudhan, Paula I. Gonzalez-Ericsson, Luigi Formisano, Nisha Unni, Shahbano Shakeel, James Z. Zhu, Khushi Ahuja, Lei Guo, María Rosario Chica-Parrado, Yuki Matsunaga, Pamela Luna, Chang-Ching A. Lin, Yasuaki Uemoto, Kyung-Min Lee, Hongli Ma, Nathaniel J. Evans, Alberto Servetto, Saurabh Mendiratta, Spencer D. Barnes, Roberto Bianco, Yisheng V. Fang, Lin Xu, Jeon Lee, Tao Wang, Justin M. Balko, Gordon B. Mills, Marilyne Labrie, Ariella B. Hanker, Carlos L. Arteaga

**Affiliations:** 1 UT Southwestern Simmons Comprehensive Cancer Center, Department of Internal Medicine, University of Texas Southwestern (UTSW) Medical Center, Dallas, Texas, USA.; 2 Department of Clinical Medicine and Surgery, University of Naples Federico II, Naples, Italy.; 3 Division of Pediatric Gastroenterology, Hepatology and Nutrition, Cincinnati Children’s Hospital Medical Center, Cincinnati, Ohio, USA.; 4 Department of Pediatrics, University of Cincinnati, Cincinnati, Ohio, USA.; 5 Departments of Medicine, Vanderbilt-Ingram Cancer Center, Vanderbilt University Medical Center (VUMC), Nashville, Tennessee, USA.; 6 Department of Medical Oncology,; 7 Quantitative Biomedical Research Center, Peter O’Donnell Jr. School of Public Health, and; 8 Lyda Hill Department of Bioinformatics, UTSW Medical Center, Dallas, Texas, USA.; 9 Department of Life Sciences, College of Natural Science, Hanyang University, Seoul, South Korea.; 10 Knight Cancer Institute, and; 11 Division of Bioinformatics and Computational Biology, Department of Medical Informatics and Clinical Epidemiology, Oregon Health and Science University, Portland, Oregon, USA.; 12 Department of Pathology, UTSW Medical Center, Dallas, Texas, USA.; 13 Department of Pathology, Microbiology and Immunology, Vanderbilt-Ingram Cancer Center, VUMC, Nashville, Tennessee, USA.; 14 Division of Oncological Sciences, Knight Cancer Institute, Oregon Health and Science University, Portland, Oregon, USA.; 15 Department of Immunology and Cell Biology, Faculty of Medicine and Health Science, Université de Sherbrooke, Sherbrooke, Quebec, Canada.; 16 Centre de Recherche du Centre Hospitalier de l’Université de Sherbrooke, Sherbrooke, Quebec, Canada.; 17 Institut de Recherche sur le Cancer de l’Université de Sherbrooke, Sherbrooke, Canada.

**Keywords:** Immunology, Oncology, Breast cancer, Chemokines, Drug therapy

## Abstract

The role of the tumor immune microenvironment (TIME) in modulating responses to antiestrogen therapy in hormone receptor–positive (HR^+^) breast cancers remains unclear. We analyzed pre- and on-treatment biopsies from patients with HR^+^ breast cancer treated with letrozole to induce estrogen deprivation (ED). Stromal tumor–infiltrating lymphocytes, assessed by H&E staining, and immune-related gene sets, including IFN-γ signaling genes, measured by RNA-Seq, were increased in ED-resistant tumors. Cyclic immunofluorescence and spatial transcriptomics revealed an abundance of CD8^+^ T cells and enhanced antigen processing and immune gene signatures in ED-resistant tumors. In this group, the expression of *CXCL9*, *CXCL10*, and *CXCL11* — chemokine genes involved in CD8^+^ T cell recruitment — and the *CXCR3* receptor were upregulated both before and after letrozole treatment. CXCL11 levels were higher in conditioned media from HR^+^ breast cancer cells cocultured with CD8^+^ T cells. Both recombinant CXCL11 and coculture with CD8^+^ T cells promoted MCF7 and T47D cell growth in estrogen-free conditions. Finally, deletion combined with silencing of the CXCL11 receptors CXCR3 and CXCR7 in MCF7 cells impaired proliferation in response to exogenous CXCL11 and to coculture with CD8^+^ T cells in estrogen-free conditions. These findings suggest that CD8^+^ T cell–associated CXCL11 in the TIME modulated the response of HR^+^ breast cancer cells to estrogen suppression.

## Abstract 2

CD8+ T cells in the tumor immune microenvironment promote resistance to estrogen suppression in HR+ breast cancer via CXCL11 and immune-related pathways.

## Graphical abstract

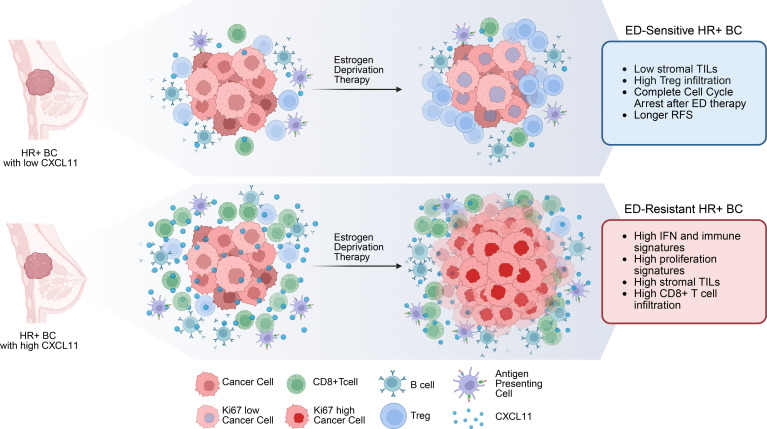

## Introduction

Almost 80% of breast cancers are hormone receptor positive (HR^+^). In these tumors, cancer cells rely on estrogen receptor (ER) signaling for survival and proliferation (1). Treatment of HR^+^ breast cancer is based on the suppression of ligand-dependent or -independent activation of ERa, which can be achieved through several approaches, such as estrogen suppression with aromatase inhibitors and selective ER degraders or modulators (2). Although these therapies have greatly improved patient survival (1), up to 20% of patients exhibit intrinsic resistance to endocrine therapy with early metastatic recurrence (2). Therefore, identifying those patients who are unlikely to benefit from estrogen deprivation (ED) and may require additional therapies is of critical importance.

Recent studies have demonstrated that the tumor immune microenvironment (TIME) may affect the outcomes of patients with human epidermal growth factor receptor 2–positive (HER2^+^) and triple-negative breast cancer (TNBC). Indeed, tumor-infiltrating lymphocytes (TILs) in these breast cancer subtypes are associated with higher rates of a pathologic complete response after neoadjuvant chemotherapy and longer patient survival (3, 4). In addition, stromal TILs in TNBC are associated with a better response to immune checkpoint inhibitors (3). In comparison, TIL numbers are generally lower in HR^+^ breast cancers (5); thus, this subtype has been considered “immunologically cold” (6). However, some reports have shown that there is a subgroup of HR^+^ breast cancers with high TIL numbers that may respond poorly to estrogen suppression (with aromatase inhibitors) and result in shorter survival times than occurs for tumors with lower TIL numbers (7–11). The molecular characteristics of such tumors have yet to be completely defined.

Cellular components in the tumor microenvironment influence each other through direct cell-to-cell contact or via soluble factors such as cytokines, chemokines (chemotactic cytokines), and growth factors. Estrogen suppression can lead to the release of several cytokines, which in turn affect the transcriptional activity of breast cancer cells (12, 13). However, a comprehensive understanding of how chemokines in the TIME shape the phenotype of HR^+^ tumors and influence the response to therapy is still lacking.

Here, we leveraged samples from a short presurgical clinical trial of early-stage, operable HR^+^ breast tumors treated with letrozole to induce ED. Tumors were classified as ED sensitive or ED resistant on the basis of the proportion of Ki67^+^ cancer cells after 2 weeks of treatment (14). Compared with ED-sensitive tumors, ED-resistant tumors exhibited higher infiltration of CD8^+^ T cells and enrichment of immune response pathways (including IFN-related signatures). Using spatial transcriptomics, we found that estrogen suppression in resistant tumors increased CD8^+^ T cell infiltration and reduced Tregs. Resistant tumors showed enrichment of antigen processing and immune gene signatures in both cancer and immune cells, which was further amplified by estrogen suppression. *CXCL9/-10/-11* mRNA expression levels were higher in ED-resistant than ED-sensitive tumors before and after treatment, and their elevated expression correlated with worse relapse-free survival (RFS) for patients with HR^+^ breast cancer. Finally, recombinant CXCL11 and CD8^+^ T cell coculture promoted HR^+^ tumor growth under ED conditions, indicating that components of the TIME directly modulated the response to endocrine therapy.

## Results

### HR^+^ breast cancers resistant to ED are enriched with aggressive molecular features and immune cell infiltration.

**Figure 1:**
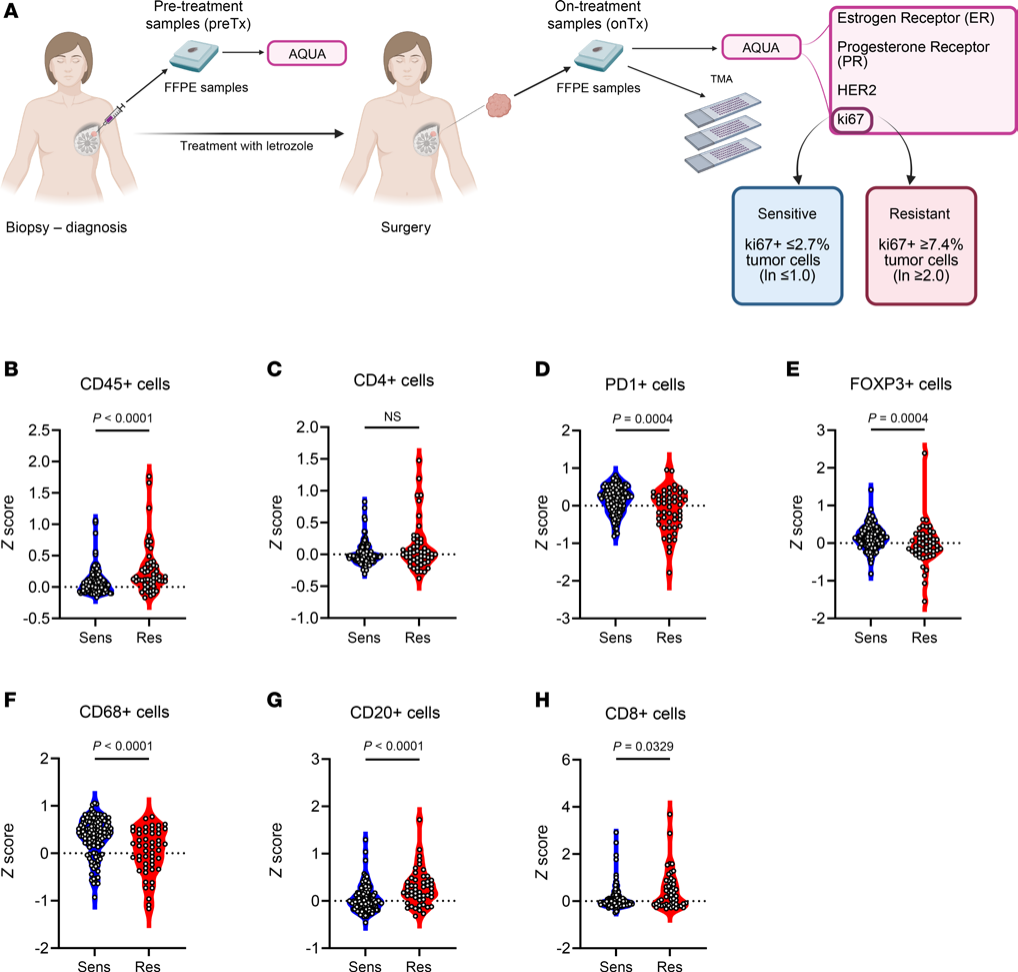
Immune cell subtypes are differentially distributed in ED-resistant versus ED-sensitive tumors. (**A**) Study design: 285 patients with stage I–III HR^+^ breast cancer were treated for 2–3 weeks with letrozole from diagnosis until surgery. Tumor samples were collected at diagnosis (pre-treatment [preTx]) and at the time of surgery (on-treatment [onTx]) and evaluated for ER, PR, HER2, and Ki67 levels. Ki67 was assessed by AQUA and/or IHC. Tumors were categorized as ED-sensitive or ED-resistant according to the Ki67 score as described in Methods. TMAs were prepared from tumor samples collected at the time of surgery. (**B**–**H**) Expression of CycIF markers in the “pressure area”: CD45 (immune cells), CD4 (CD4^+^ T cells), PD1 (expressed mainly by exhausted immune cells), FOXP3 (expressed by regulatory cells, such as Tregs), CD68 (macrophages), CD20 (B cells), and CD8 (CD8^+^ T cells). *n* = 124 ED-sensitive (sens) samples, *n* = 50 ED-resistant (res) samples. The Wilcoxon rank-sum test was applied to compare cell-type distributions, and *P* values are reported in the figure panels.

For this study, we used evaluable biospecimens collected prospectively from postmenopausal women with newly diagnosed stage I–III HR^+^ breast cancer, who consented to enroll in a short presurgical clinical trial (Figure 1A). Patient characteristics are shown in Table 1. In all samples, ER, progesterone receptor (PR), HER2, and Ki67 levels were assessed following established protocols (Supplemental Figure 1A; see Methods; supplemental material available online with this article; https://doi.org/10.1172/JCI188458DS1). Ki67 scores evaluated from the surgical samples (hereafter to referred to as on-treatment or onTx) were used to categorize tumors as sensitive to ED (ED-sensitive, Ki67 ≤2.7% tumor cells) or resistant (ED-resistant, Ki67 ≥7.4%), following the tertiles used in the IMPACT trial (14). In the overall ED-resistant group, PR levels were significantly downregulated (*P* < 0.0001). However, this effect was not statistically significant in the subset of patients whose tumors showed an increase or no change in Ki67 levels upon treatment (Supplemental Figure 1B).

ED-resistant tumors expressed lower baseline levels of the ER and PR (Supplemental Figure 1, C and D), features correlated with a worse response to endocrine treatment (15). Next, we performed RNA-Seq on 86 pre-treatment and 87 on-treatment samples. First, we inferred the risk of recurrence (ROR) in pre-treatment biopsies using a previously described method (16). The ROR score, initially developed by Parker et al. (17), is a prognostic tool for postmenopausal women with HR^+^HER2^–^ tumors. Women with an ROR score below 40 were considered at low risk and those with a score above 60 were considered at high risk of relapse. Patients with sensitive tumors had a median ROR score of 26.38, whereas those with resistant tumors had a median ROR score of 67.48 (Supplemental Figure 1E, *P* < 0.0001). The prediction analysis of microarray 50 (PAM50) subtype of 81 matched pre-treatment and on-treatment samples showed a different distribution of luminal A and luminal B cancers at baseline within ED-sensitive versus ED-resistant tumors (Supplemental Figure 1, F and G). After 2 weeks of treatment, almost all ED-sensitive tumors shifted toward less aggressive subtypes (normal-like or luminal A), however, this was not the case in the ED-resistant group (Supplemental Figure 1, F and G).

Next, we asked what molecular pathways are differentially upregulated in ED-resistant tumors versus ED-sensitive tumors. Gene set enrichment analysis (GSEA) in on-treatment samples showed that the G_2_M checkpoint (normalized enrichment score [NES] = 2.75), E2F targets (NES = 2.72), MYC targets (NES = 2.64), and mitotic spindle (NES = 1.86) pathways were upregulated in ED-resistant versus ED-sensitive tumors (Supplemental Figure 2A), consistent with the higher tumor cell proliferation in ED-resistant tumors. Immune-related signatures, including IFN-α response, IFN-γ response, and allograft rejection, were the top enriched gene sets that were increased in ED-resistant tumors (Supplemental Figure 2B). To complement these findings, we assessed the stromal TIL score in H&E-stained tumor sections (7). The average stromal TIL score in the entire cohort of pre-treatment tumors was 3.8% and in the on-treatment tumors was 4.90% (data not shown). However, when stratified by response to letrozole, the average stromal TIL score in the pre-treatment samples was 2.44% in the ED-sensitive group compared with 7.23% in the ED-resistant group (*P* = 0.0013, Supplemental Figure 2C). In the treated samples, the average stromal TIL score was 2.75% in ED-sensitive tumors versus 10.7% in ED-resistant tumors (*P* = 0.0001, Supplemental Figure 2D).

Taken together, these data define the characteristics of a subgroup of patients with HR^+^ breast tumors who responded poorly to estrogen suppression, retained high tumor cell proliferation, and exhibited enrichment in immune-related pathways, higher stromal TIL scores, and a worse survival probability (i.e., higher ROR scores).

### The TIME of ED-resistant tumors exhibits an immune-inflamed phenotype.

The activation of immune-related gene signatures and upregulation of stromal TILs in ED-resistant tumors (Supplemental Figure 2, A–D) suggested a link between resistance to ED and the TIME. To delve deeper into the role of the TIME in modulating the response of breast cancer to endocrine therapy, we conducted a comprehensive analysis of intratumoral immune cell infiltration. Stromal TILs are located in the stroma among the cancer cell clusters, lacking direct contact with cancer cells. In contrast, intratumoral TILs are in the immediate proximity of cancer cells (18). To investigate the intratumoral TIME, we prepared tissue microarrays (TMAs) from formalin-fixed, paraffin-embedded (FFPE) sections of on-treatment specimens and analyzed them using cyclic immunofluorescence (CycIF) with 38 antibodies recognizing proteins involved in proliferation, hormone receptors, and immune cell markers (Supplemental Table 1 and Supplemental Figure 3A). CycIF performs highly multiplexed IF imaging, preserving cellular spatial organization through multiple cycles of tissue staining (19). E-cadherin and/or pan-cytokeratin (PanCK) markers were used to identify and label cancer cells. Spatial enrichment of immune cells was assessed by quantifying the expression of immune markers in the area within a 2-cell radius of each labeled tumor cell, designated as the “pressure area” (Supplemental Figure 3B, see Methods). We found that cells positive for CD45 (a marker for all immune cells) were more abundant in the TIME of ED-resistant compared with ED-sensitive tumors (Figure 1B, *P* < 0.0001). Moreover, the TIME of ED-resistant tumors exhibited enrichment of CD8^+^ T cells (*P* = 0.033) and CD20^+^ B cells (*P* < 0.0001), whereas the TIME of ED-sensitive tumors was enriched with PD1^+^ cells (*P* = 0.0004), CD68^+^ macrophages (*P* < 0.0001), and FOXP3^+^ cells (*P* = 0.0004) (Figure 1, C–H). Finally, to infer the status of the CD8^+^ T cells, we evaluated the expression of several genes associated with cytolytic activity and CD8^+^ T cell activation (20) and found that ED-resistant tumors had higher scores than did ED-sensitive tumors (Supplemental Figure 3C).

In summary, ED-resistant tumors were characterized by a TIME enriched with CD8^+^ T and CD20^+^ B cells and higher cytolytic activity, suggesting potential antitumor immune activity.

### Antigen processing and T cell immunity pathways are upregulated in the TIME of ED-resistant tumors.

**Figure 2:**
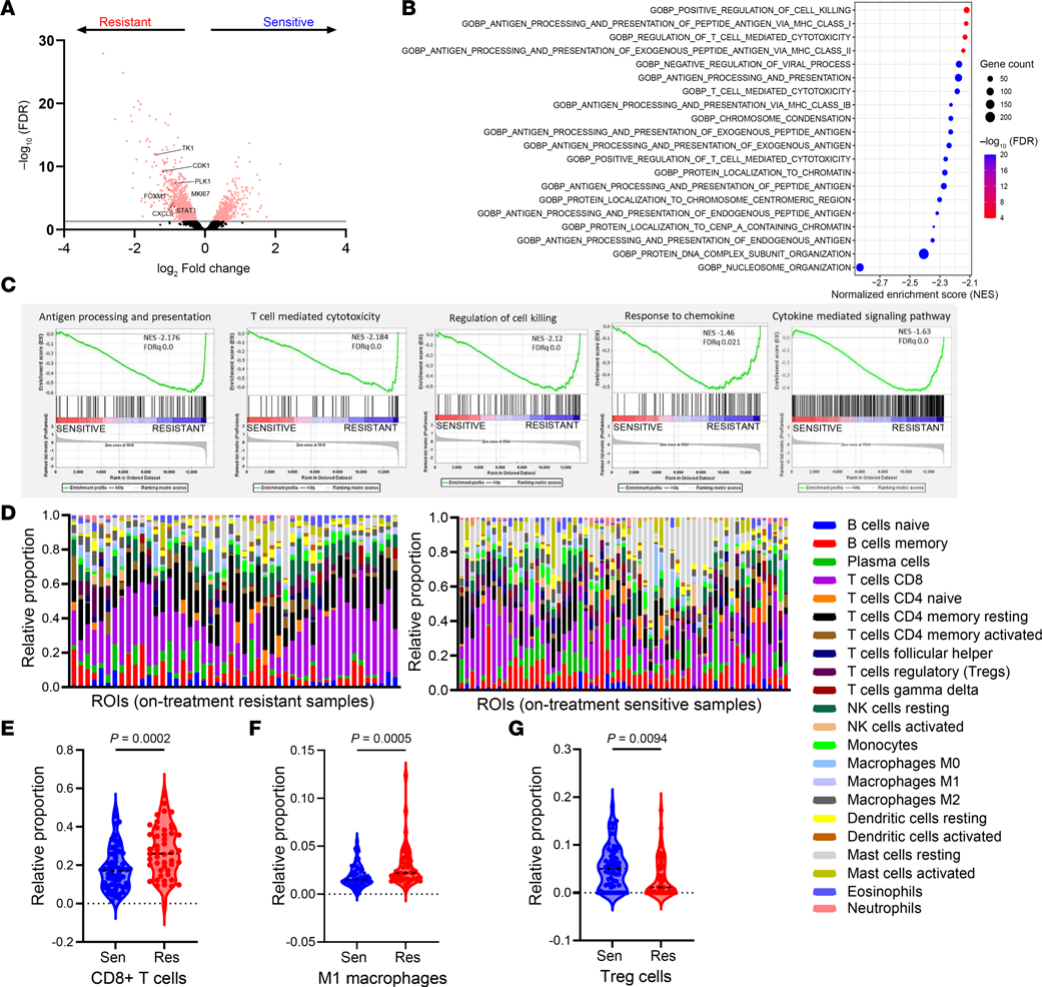
T cell immunity pathways and CD8^+^ T cells are enriched in on-treatment, ED-resistant tumors. (**A**) Volcano plot of the genes differentially expressed in cancer cells of on-treatment, ED-resistant tumors. (**B**) Gene Ontology biology process analysis using the genes in **A**. (**C**) Selected GSEA from the list in **B**. (**D**) CIBERSORT bar chart in ED-resistant and ED-sensitive on-treatment tumors, showing the 22 immune cell subtypes distribution in each ROI. (**E**–**G**) Violin plots comparing the distribution of CD8^+^ T cells, M1 macrophages, and Tregs from **D** in ED-sensitive tumors (ROI *n* = 61) versus ED-resistant tumors (ROI *n* = 48). The Wilcoxon rank-sum test was applied; *P* < 0.05 was considered statistically significant.

To investigate the changes in the transcriptome of cancer and immune cells, we selected 20 tumor samples (8 pre-treatment and 12 on-treatment) from a second cohort of patients and performed spatial transcriptomics profiling using the NanoString GeoMx platform (Supplemental Figure 4). We collected cell-type–specific spatial transcriptomics data from cancer cells (PanCK^+^) and immune cells (CD45^+^). First, we compared the transcriptomes of cancer cells in on-treatment ED-resistant and ED-sensitive tumors (Figure 2A). Gene Ontology analysis showed that the top 40 upregulated pathways in ED-resistant tumors were primarily related to proliferation signatures, antigen-processing and presentation machinery (APM), and T cell–related immunity (Figure 2, B and C). Comparison of the transcriptomes of immune cells revealed that the top upregulated pathways in ED-resistant tumors were associated with T cell immunity (proliferation and cytotoxicity) and the APM (Supplemental Figure 5, A and B). We then used CIBERSORT (21) to estimate the fraction of 22 immune cells within the CD45^+^ cell population of on-treatment tumors (Figure 2D). We found several differences in the distribution of the immune cell subtypes (Supplemental Figure 5C). Among these, CD8^+^ T cells (Figure 2E, *P* < 0.001) and IFN-γ–activated M1 macrophages (Figure 2F, *P* < 0.001) were significantly enriched in ED-resistant tumors, whereas Tregs were enriched in ED-sensitive tumors (Figure 2G, *P* < 0.01).

Collectively, these analyses suggest that both cancer and immune cell compartments in ED-resistant tumors upregulated gene expression pathways involved in antitumor immune activity upon ED with aromatase inhibitors. In agreement, CD8^+^ T cells were enriched in ED-resistant tumors, together with IFN-regulated, M1-like macrophages, whereas FOXP3^+^ Tregs were enriched in ED-sensitive tumors.

### Estrogen suppression reshapes cancer and immune cell compartments in ED-resistant tumors.

**Figure 3:**
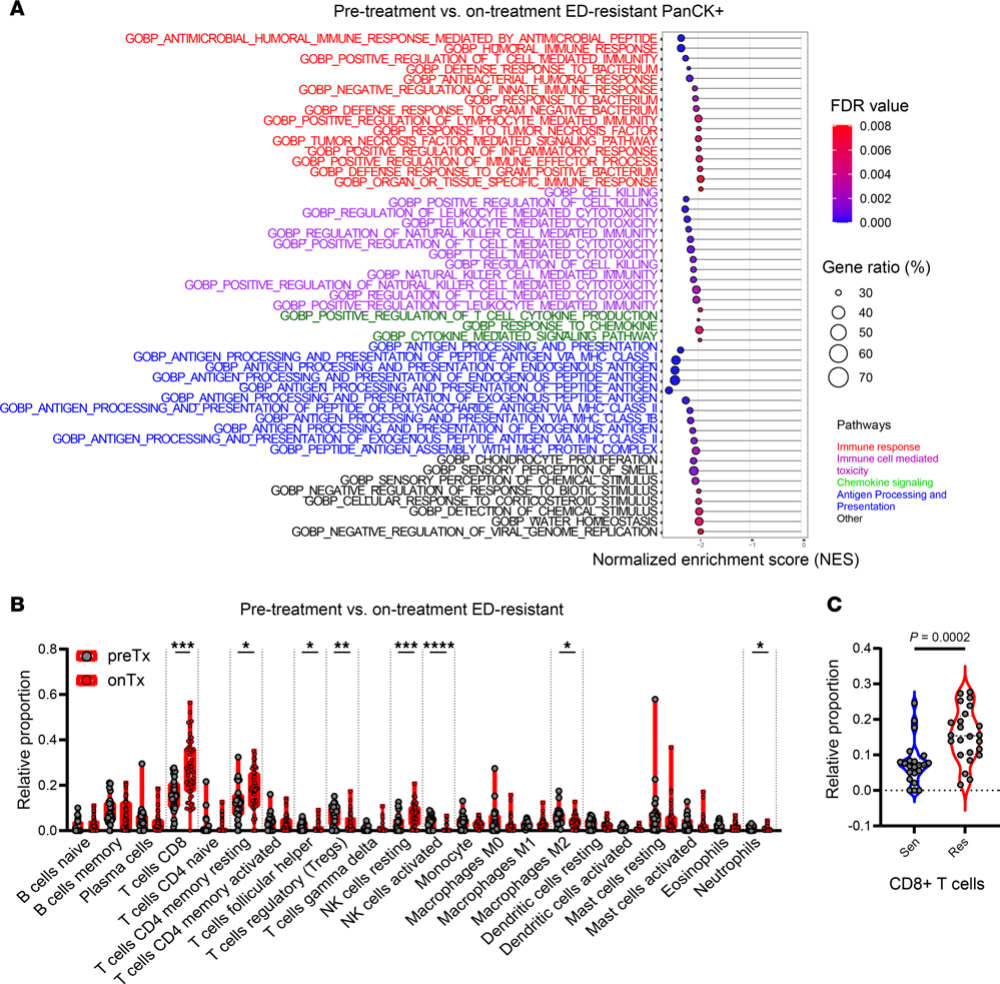
Endocrine treatment reshapes the TIME in ED-resistant tumors. (**A**) List of the top pathways enriched in cancer cells in ED-resistant tumors upon estrogen suppression. (**B**) Comparison of immune cell distribution estimated using the CIBERSORT method, comparing pre-treatment (ROI *n* = 24) versus on-treatment (ROI *n* = 48) ED-resistant tumors. (**C**) Violin plot comparing the estimated distribution of CD8^+^ T cells in pre-treatment–sensitive (ROI *n* = 22) versus pre-treatment–resistant tumors (ROI *n* = 24). *P < 0.05, **P < 0.01, ***P < 0.001, and ****P < 0.0001, by Wilcoxon rank-sum test for comparison of cell-type distributions. *P* < 0.05 was considered statistically significant.

To investigate letrozole-induced changes in immune-related pathways within cancer and immune cell populations, we compared spatial transcriptomics profiles of pre-treatment versus on-treatment tumors in both the ED-sensitive and ED-resistant groups (example in Supplemental Figure 4). In PanCK^+^ cancer cells from ED-resistant tumors, letrozole treatment upregulated pathways related to the APM, the immune response, T cell– and NK cell–mediated cytotoxicity, and cytokine signaling (Figure 3A). In contrast, none of these pathways was modulated by letrozole in ED-sensitive tumors (Supplemental Table 2). Similarly, transcriptomics analysis of the CD45^+^ cells revealed broad upregulation of pathways related to the APM, the immune response, and T cell– and NK cell–mediated cytotoxicity following letrozole treatment, with stronger upregulation observed in ED-resistant than in ED-sensitive tumors (Supplemental Figure 6A and Supplemental Table 3). CIBERSORT deconvolution of CD45^+^ cells transcriptomes from ED-resistant tumors further showed a significant increase in CD8^+^ T cells (*P* < 0.001) and a reduction of Tregs (*P* < 0.01) and M2-like macrophages (*P* < 0.05) upon estrogen suppression (Figure 3B). Finally, we performed similar pathway analyses, comparing pre-treatment ED-sensitive and pre-treatment ED-resistant tumors (Supplemental Tables 4–7). Notably, CIBERSORT deconvolution of pre-treatment tumors (Supplemental Figure 6B) showed that ED-resistant tumors already exhibited CD8^+^ T cell enrichment at diagnosis, prior to therapy (Figure 3C, *P* < 0.001).

These data suggest that, in ED-resistant tumors, estrogen suppression can alter the cell-type distribution and gene expression of immune cells in the breast TIME, even though this suppression does not inhibit the proliferation of HR^+^ cancer cells. Moreover, the preexisting enrichment of CD8^+^ T cells in resistant tumors raises the question of whether these cells play a causal role in resistance to antiestrogen therapy.

### CD8^+^ T cells and associated chemokines directly promote estrogen-independent growth of HR^+^ breast cancer cells.

**Figure 4:**
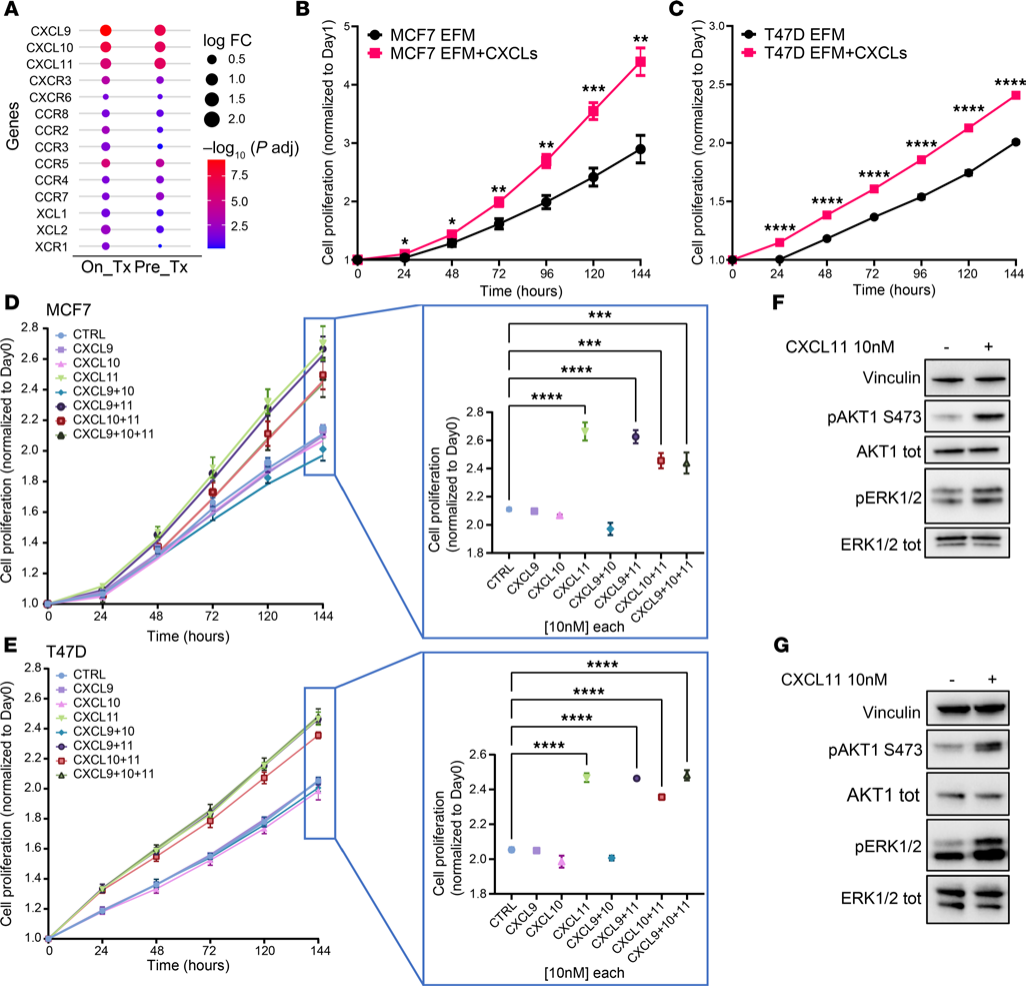
CXCL11 promotes resistance to estrogen suppression. (**A**) Expression of chemokines associated with antitumor activity upregulated in ED-resistant (versus. ED-sensitive) tumors after (on the left) and prior to (on the right) treatment with letrozole. *P* adj, adjusted *P* value. (**B** and **C**) Growth curves of MCF7 (**B**) or T47D (**C**) cells, seeded in EFM, alone or in the presence of CXCL9, CXCL10, and CXCL11, acquired using IncuCyte live cells. The mean ± SD of the number of cells is shown (note that SDs are not always visible when smaller than the size of the symbol). *P < 0.05, **P < 0.01, ***P < 0.001, and ****P < 0.0001, by unpaired *t* test. Experiments were performed at least 3 times in triplicate wells. (**D** and **E**) MCF7 (**D**) or T47D (**E**) cell proliferation evaluated by IncuCyte, as in **B** and **C**. Cells were seeded as in **B** and **C** and treated with PBS plus 1% BSA (control) or with CXCL9, CXCL10, and CXCL11 (10 nM each) or double (CXCL9 + CXCL10, CXCL9 + CXCL11, CXCL10 +CXCL11) or triple (CXCL9 + CXCL10 + CXCL11) combinations. On day 5, all the treatment arms were compared with vehicle using 1-way ANOVA with Dunnett’s correction for multiple testing; *P* < 0.05 was considered statistically significant (*P < 0.05, **P < 0.01, ***P < 0.001, and ****P < 0.0001). Experiments were performed at least 3 times in triplicate wells. (**F** and **G**) Immunoblot analysis of MCF7 (**F**) and T47D (**G**) levels in cell lysates. Cells were seeded overnight in serum-starved medium, and the lysates were collected 15 minutes after adding 10 nM CXCL11 and then probed with the indicated antibodies. tot, total.

Immune cell recruitment to cancer sites occurs through chemotactic cytokines. These are soluble factors released into the tumor microenvironment, where they mediate cell-to-cell communication. Thus, we speculated that cytokines in the TIME may play a role in the differential response to endocrine therapy in HR^+^-sensitive versus -resistant tumors. GSEA of RNA-Seq data showed that cytokine/chemokine-related pathways were upregulated in ED-resistant versus ED-sensitive tumors (Figure 2C and Figure 3A). In particular, ED-resistant tumors exhibited upregulation of *CXCL9*, *CXCL10*, and *CXCL11*, as well as their receptor *CXCR3*, in both on- and pre-treatment biopsies (Figure 4A). Upon letrozole treatment, the expression of *XCL1* and *XCL2* chemokines and their receptor *XCR1* was enhanced in ED-resistant tumors (Figure 4A).

Next, we examined whether high expression of *CXCL9*, *CXCL10*, and *CXCL11* in ED-resistant tumors was causally associated with their estrogen-independent phenotype. First, we interrogated publicly available datasets (Kaplan-Meier Plotter; kmplot.com, see Methods) and found that high expression of *CXCL9*, *CXCL10*, and *CXCL11* correlated with shorter RFS for patients with HR^+^ breast cancer treated with endocrine therapy compared with those with low expression (*CXCL9*, 76.02 vs. 127.8 months, HR 1.39, log-rank *P* = 0.01; *CXCL10*, 72.49 vs. 139.5 months, HR 1.79, log-rank *P* = 9.4 × 10^–6^; *CXCL11*, 75.9 vs. 128.7 months, HR 1.74, log-rank *P* = 4.4 × 10^–5^) (Supplemental Figure 7, A–C). We then treated 2 HR^+^ breast cancer cell lines, MCF7 and T47D, with recombinant CXCL9, CXCL10, and CXCL11. All experiments were conducted in estrogen-free medium (EFM) to phenocopy letrozole-induced estrogen suppression in patients in the clinical trial. Chemokine-treated cells proliferated at a significantly faster rate compared with controls (Figure 4, B and C). Similar results were obtained in the HR^+^ ZR-75-1 and HCC1428 cell lines (Supplemental Figure 7, D and E). After ranking the effects of the chemokines alone or in combination, CXCL11 emerged as the most potent cytokine to stimulate the estrogen-independent growth of MCF7 and T47D cells (Figure 4, D and E). CXCR3 and CXCR7/ACKR3 are key receptors mediating CXCL11 activity (22). After confirming their expression in both cell lines by immunoblot analysis and reverse transcription quantitative PCR (RT-qPCR) (Supplemental Figure 7, F and G), we investigated the effect of CXCL11 on the downstream pathways. Addition of recombinant CXCL11 induced phosphorylated AKT (pAKT) and pERK in both MCF7 and T47D cells (Figure 4, F and G), consistent with its effect on survival under estrogen-deprived conditions. We used CRISPR/Cas9 to knock out *CXCR3* or *CXCR7* in MCF7 cells (Supplemental Figure 8A). *CXCR3* knockout modestly impaired cell proliferation and greatly reduced the cells’ response to CXCL11 stimuli (Supplemental Figure 8B). In addition, knockout of *CXCR7* markedly impaired cell proliferation, and adding CXCL11 minimally rescued this effect (Supplemental Figure 8C). On the basis of these findings, we suspected a partially redundant role for CXCR3 and CXCR7. Thus, we transduced siRNAs against *CXCR7* in *CXCR3*-knockout cells and siRNAs against *CXCR3* in *CXCR7*-knockout cells and treated cells with exogenous CXCL11 (Supplemental Figure 8, B and C). Simultaneous downregulation of both receptors markedly impaired baseline and CXCL11-stimulated MCF7 cell proliferation.

**Figure 5:**
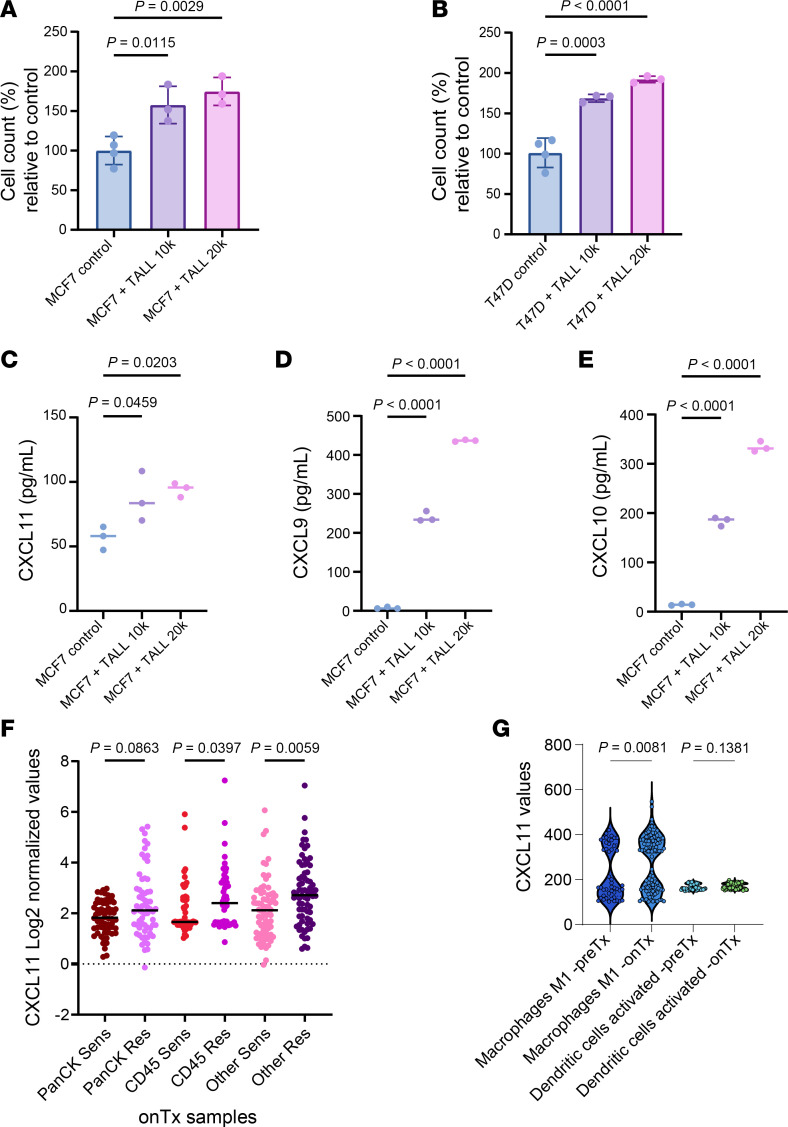
Coculture with CD8^+^ T cells promotes estrogen-independent growth of HR^+^ breast cancer cells. (**A** and **B**) MCF7 (**A**) and T47D (**B**) cells with or without T-ALL-104 cells in Transwell inserts were seeded in EFM as described in Methods. Data represent the mean ± SD of MCF7, and T47D cell numbers were evaluated 6 days after seeding. Experiments were performed at least 3 times in triplicate wells. Statistical differences were assessed using 1-way ANOVA with Dunnett’s correction for multiple testing; *P* < 0.05 was considered statistically significant. (**C**–**E**) ELISA analysis of CXCL11 (**C**), CXCL9 (**D**), and CXCL10 (**E**) levels in MCF7 with or without T-ALL-104 cells as measured by ELISA. MCF7 cells with or without T-ALL-104 cells were seeded in EFM as in **A** and **B**. Media conditioned by MCF7 cells with or without T-ALL-104 cells were collected after 6 days. Experiments were performed at least 3 times in triplicate wells. Data represent the mean ± SD. Statistical differences were assessed using 1-way ANOVA with Dunnett’s correction for multiple testing; *P* < 0.05 was considered statistically significant. (**F**) CXCL11 expression in PanCK^+^, CD45^+^, PanCK^–^CD45^–^ compartments evaluated using spatial transcriptomics. Kruskal-Wallis test and Benjamini-Krieger-Yekutieli FDR adjustment for multiple comparisons was applied to compare cell-type distributions; *q* < 0.05 was considered statistically significant. (**G**) CXCL11 expression inferred from CIBERSORT. Kruskal-Wallis test and Benjamini-Hochberg FDR adjustment for multiple comparisons was applied to compare CXCL11 expression across cell types and time points; *q* < 0.05 was considered statistically significant.

We next tested whether HR^+^ breast cancer cells would proliferate better in the presence of cocultured CD8^+^ T cells, as suggested by spatial transcriptomics analysis. To test this, we designed a coculture system in which T-ALL-104 (CD8^+^ T) cells could not interact with MCF7 or T47D cells, while sharing their growth medium (Supplemental Figure 9A, see Methods). MCF7 and T47D cells cocultured with T-ALL-104 cells exhibited 1.5- to 2-fold faster proliferation compared with cells seeded without T-ALL-104 cells (Figure 5, A and B). This effect was not observed in cocultured MCF7 cells with combined gene deletion and RNA silencing of *CXCR3* and *CXCR7* (Supplemental Figure 9, B and C). A cytokine ELISA of coculture conditioned media showed that CXCL11 levels increased 1.5- to 2.0-fold after 6 days compared with conditioned media from MCF7 cells in the absence of T-ALL-104 cells (Figure 5C). Similar results were observed with CXCL9 and CXCL10 in the coculture conditioned media (Figure 5, D and E).

In addition to CD8^+^ T cells, CXCL11 may be released by other cell types in vivo. To explore this, we interrogated the spatial transcriptomics data for CXCL11 expression distribution across compartments and found a diffuse increase in the ED-resistant tumors (Figure 5F). We then analyzed CXCL11 expression distribution across the 22 immune cell types deconvoluted using CIBERSORT in all pre-treatment and on-treatment samples. We found that only 2 cell types had imputable expression of CXCL11: M1 macrophages and activated DCs (Figure 5G).

Finally, we investigated whether CXCL11 levels are predictive of a poorer response to estrogen suppression therapy. Using RNA-Seq data from patients enrolled in the clinical trial, we developed the receiver operating characteristic (ROC) curve for CXCL11, which showed an AUC of 0.777 (Supplemental Figure 9D). Consistent with these results, when categorizing the variables, *CXCL11* gene expression levels were higher in the ED-resistant group and lower in the ED-sensitive group (*P* < 0.0001; Supplemental Figure 9E). Taken together, these data suggest that HR^+^ breast cancers can hijack T cell–recruiting chemokines to bypass the effects of therapeutic ED. This also suggests that high CXCL11 expression could be used as a biomarker to predict resistance to endocrine therapy.

## Discussion

In this study, we show that a subset of HR^+^ breast tumors with aggressive clinical features, including resistance to estrogen suppression, exhibited a TIME enriched with CD8^+^ T cells and CXCL11. Coculture with CD8^+^ T cells promoted estrogen-independent growth of HR^+^ breast cancer cells. This effect was associated with increased levels of CXCL11 in conditioned media. In line with this finding, treatment with recombinant CXCL11 enabled HR^+^ breast cancer cells, typically sensitive to estrogen suppression, to overcome hormone dependence. This effect was abrogated by deletion and silencing of the CXCL11 receptors CXCR3 and CXCR7. Together, these findings support a causal role for the CXCL11 axis in mediating antiestrogen resistance in HR^+^ breast tumors with a CD8^+^ T cell–enriched microenvironment.

In this study, we classified HR^+^ breast cancers as ED sensitive or ED resistant on the basis of their Ki67 score upon letrozole-induced estrogen suppression: ED-sensitive cancers were defined as having 2.7% or less Ki67^+^ tumor cells and ED-resistant cancers 7.4% or greater Ki67^+^ tumor cells, as in the IMPACT trial (23). We found that PR levels were downregulated in both ED-sensitive and ED-resistant tumors (Supplemental Figure 1B), suggesting that, while letrozole treatment effectively inhibited ligand-dependent ER signaling, a subset of tumors exhibited a disconnect between estrogen signaling and proliferation, warranting further investigation.

We found that several features were associated with worse prognosis in the ED-resistant subgroup, including immune-related hallmarks. Immune-related signatures were also associated with poor responses to neoadjuvant endocrine treatment in a recent study (11). In addition, we found a distinct PAM50 subtype changes toward a more aggressive molecular subtype (Supplemental Figure 1, F and G). Of note, approximately 34% of ED-sensitive tumors were luminal B at baseline, yet nearly all transitioned to normal-like or luminal A subtype after short-term treatment with letrozole. By contrast, several ED-resistant tumors shifted toward more aggressive subtypes (e.g., normal to basal, luminal A to HER2-enriched). These observations raise the possibility that assessing PAM50 subtype switching after short-term endocrine therapy could provide predictive insights and merits further exploration.

HR^+^ breast cancer has generally been considered to reside in an “immune-cold” microenvironment. Recent evidence suggests that the TIME of HR^+^ breast cancers is primarily composed of immunosuppressive macrophages (24) and typically contains lower numbers of stromal TILs than other subtypes (5). Consistent with this, our clinical trial data showed that ED-sensitive tumors exhibited increased infiltration of CD68^+^ macrophages (Figure 1F), with an average score for stromal TILs of 2.44% in untreated tumors and 2.75% in on-treatment tumors (Supplemental Figure 2, C and D). ED-resistant tumors, however, contained a significantly higher number of stromal TILs, with an average score of 7.23% and 10.7% in pre-treatment versus on-treatment tumors, respectively (Supplemental Figure 2, C and D). These findings align with previous reports, in which stromal TILs have been associated with endocrine resistance and worse survival rates for HR^+^ breast cancer. In contrast, elevated numbers of TILs have been associated with a better prognosis in HER2^+^ and TNBC (7–10).

To better understand the role of the immune microenvironment in the context of ED, we evaluated the immune cell types present in the “pressure area” surrounding cancer cells (Supplemental Figure 3B). We found that ED-resistant tumors had higher CD8^+^ T cell infiltration in on-treatment samples using both CycIF (Figure 1H) and the CIBERSORT deconvolution algorithm of spatial transcriptomics data (Figure 2E); this infiltration was further enhanced by estrogen suppression (Figure 3B). These results suggest that, among several immune cell types included in the TIME, CD8^+^ T cells were strongly associated with ED resistance.

We speculated that cytokines in the TIME play a role in response to antiestrogen therapy. Indeed, gene expression studies showed upregulation of *CXCL9*, *CXCL10*, and *CXCL11* in ED-resistant tumors, both before and after estrogen suppression (Figure 4A), and these chemokines are known to be involved in CD8^+^ T cell and NK cell recruitment (25). To expand upon this finding, we developed a coculture system in which HR^+^ breast cancer and CD8^+^ T cells shared the same culture medium, but a physical barrier impeded T cell–induced cytotoxicity (Supplemental Figure 9A). In the absence of estradiol, HR^+^ breast cancer cells grew faster in the presence of cocultured CD8^+^ T cells (Figure 5, A and B). This finding is of particular interest because it points to a dual role of immune cells in HR^+^ breast cancer. Although immune cells, and T cells in particular, are generally expected to exert antitumor effects, our data suggest that they may also contribute to tumor persistence and resistance to therapy.

CXCL11 levels were elevated in conditioned media from cocultures of breast cancer/CD8^+^ T cells (Figure 5C), in agreement with gene expression data from ED-resistant tumors (Figure 4A). In line with this finding, exogenous CXCL11 and cocultures with CD8^+^ T cells were sufficient to sustain estrogen-independent growth of HR^+^ breast cancer cells (Figure 4, D–G). This phenotype was lost when CXCR3/-7 receptors were removed through combined knockout and knockdown (Supplemental Figure 8 and Supplemental Figure 9, B and C). These data suggest that CXCL11 in the TIME may, at least in part, be causally associated with the sustained proliferation of letrozole-treated primary tumors that we observed in samples from the clinical trial. In addition, CXCL11 may serve as a biomarker of an immune-active microenvironment that can be targeted therapeutically.

To achieve an effective antitumor immune response, (a) cytotoxic T cells must be recruited to the tumor site and undergo proper activation, and (b) the APM, including DCs and MHC complexes, must be engaged (25, 26). We found that elements of these conditions were present in ED-resistant tumors. Moreover, after treatment with ED, we found increased expression of XCL1 and XCL2, chemokines secreted by activated CD8^+^ T cells. These chemokines interact with XCR1 expressed on conventional DC type 1 (cDC1), a population of DC cells with the unique capacity to cross-present antigens to CD8^+^ T cells (27). This interaction induces an effective cytotoxic T lymphocyte response (27). Despite this, the immune system fails to control the growth of ED-resistant tumors.

Notably, the enhanced immune activity observed in these tumors may represent a therapeutic opportunity. Indeed, recent clinical trials have shown an increased rate of pathological complete responses in high-risk HR^+^ breast cancers treated with neoadjuvant immune checkpoint inhibitors (28–30). Preliminary data presented at the American Society of Clinical Oncology (ASCO) 2024 meeting by the FLEX investigators (31) showed that patients with improved responses to immunotherapy showed a heightened immune active state, including high infiltration of CD8^+^ T cell and B cells, elevated MHC II expression, and the presence and activation of antigen-presenting cells — all features that characterize the patients with endocrine resistance in the present study. These observations further suggest that baseline levels of CXCL11 could serve as a potential biomarker to identify patients with endocrine-resistant tumors who may respond to immune checkpoint inhibitors.

We recognize that, in addition to CD8^+^ T cells, other cells may be contributing to CXCL11 in the TIME, such as other immune cells, endothelial cells, fibroblasts, or cancer cells themselves. Moreover, our initial discovery with CycIF used a limited number of antibodies. Thus, other TIME-derived factors that also contribute to estrogen-independent proliferation of breast cancer cells could have been missed. In an attempt to identify where CXCL11 may be released in the TIME, we interrogated the GeoMx expression data and found a generalized increase in all compartments (PanCK^+^, CD45^+^, PanCK^–^CD45^–^) of the ED-resistant tumors (Figure 5F). Specifically, in our cohort, M1 macrophages and activated DCs were the primary immune cell subtypes contributing to *CXCL11* expression (Figure 5G).

In summary, our transcriptomics analysis revealed a subset of patients with HR^+^ breast tumors harboring a distinct intrinsic biology, defined by estrogen-independent proliferation — as evidenced by the absence of a letrozole effect on proliferation markers and signatures — and a TIME that may be exploitable using new therapeutic strategies. Our findings also highlight a role for CD8^+^ T cell–associated CXCL11 in promoting resistance to estrogen suppression in HR^+^ breast cancer. CXCL11 is linked to a TIME enriched with transcriptional features of antitumor immune activity, suggesting its potential as a biomarker to guide patient selection for immune-focused therapies.

## Methods

### Sex as a biological variable

In this study, we exclusively used samples collected from female patients, as the clinical trial was designed to investigate the role of estrogen suppression in postmenopausal women. We cannot draw any conclusion as to whether our findings apply to male breast cancer.

### Study design

Two hundred twenty-four postmenopausal women diagnosed with stage I–III HR^+^ HER2^–^ breast cancer at the VUMC and 61 at the UTSW Simmons Comprehensive Cancer Center were enrolled in a short, presurgical clinical trial, in which the primary endpoint was the Ki67 score on the surgical biopsy, after 2–3 weeks of treatment with letrozole (2.5 mg/day) (NCT00651976) (32–35). Patient demographics are shown in Table 1. In the first cohort (VUMC), the trial was performed before 2015 (prior to NIH’s NOT-OD-15-089; https://grants.nih.gov/grants/guide/notice-files/not-od-15-089.html) and did not collect data on race or ethnicity. These data were collected in the second cohort (UTSW) and were self-reported by the participants. Forty-three of the patients self-reported as White (70.5%), 26 of whom self-reported as Non-Hispanic/Latino, 14 as Hispanic/Latino, and 3 did not specify; 4 patients self-reported as Asian (6.5%), 12 patients as Black (19.7%), and 2 patients did not specify their race or ethnicity (3.3%). Data on patient sample availability and use from the VUMC cohort are shown in Supplemental Figures 9–11.

Fresh tumor biopsies and/or FFPE tumor blocks were collected. From the first cohort (VUMC), 5 μm tumor sections were stained with H&E. An expert breast pathologist evaluated the presence of stromal TILs following published recommendations (18). In this first cohort, ER, PR, HER2, and Ki67 levels were evaluated using quantitative immunofluorescence automated quantitative analysis (AQUA) (36). Fluorescence multiplexed IHC was conducted at Genoptix Inc. Medical Laboratories on both the diagnostic (pre-treatment) and surgical (on-treatment) biopsies. This method utilizes multiplexed IF to delineate invasive cancer cells with tagged pan-cytokeratin and DAPI staining (nuclei) in 10–25 regions of interest (ROIs), measuring approximately 10,000 tumor nuclei (37). The Ki67 labeling index was calculated by the natural log (ln) transformation of the sum of the AQUA score and a small factor to account for samples with a score of zero [Ki67 labeling index = ln(AQUA score + 0.01)]. In the second cohort (UTSW), ER, PR, HER2, and Ki67 levels were evaluated using IHC. The correlation between the results of AQUA and IHC has been previously reported by us (38), which we confirmed in the first cohort (data not shown). Patients whose surgical biopsies showed 2.7%–7.4% Ki67^+^ cells (intermediate) were excluded from these analyses.

### RNA extraction and sequencing

RNA was extracted from 4–8 10 μm FFPE tumor sections using the Promega Maxwell 16 LEV RNA FFPE Purification Kit and instrument, according to the manufacturer’s instructions. cDNA library construction for RNA-Seq: total RNA was quantified using a Qubit (Life Technologies, Thermo Fisher Scientific), and RNA quality was assessed using an Agilent Bioanalyzer. For tumors in which RNA met quality control standards (RNA integrity number, [RIN] >7), 100 ng RNA was used for library preparation following the manufacturer’s protocol for RNA ACCESS (Illumina). Briefly, first and second strand cDNA synthesis was performed, universal adapters were ligated, and coding regions were selected by 2 consecutive hybrid captures followed by PCR enrichment. After enrichment, the libraries were quantified by qPCR using the KAPA Library Quantification Kit for Illumina Sequencing platforms, pooled and normalized to 2 nM, and denatured using 0.2 N NaOH prior to sequencing. Flowcell cluster amplification and sequencing were performed according to the manufacturer’s protocols using the HiSeq 3000. Each run was a 76 bp paired-end run with an 8-base index barcode. Data were analyzed using the Broad Picard Pipeline which includes demultiplexing and data aggregation. Alignment, quantification, and differential analysis were performed using the QBRC_BulkRnaSeqDE pipeline (https://github.com/QBRC/QBRC_BulkRnaSeqDE). Briefly, alignment of reads to the human reference genome (GRCh38, https://www.ncbi.nlm.nih.gov/assembly/GCF_000001405.26) was done using STAR (version 2.7.2b). FeatureCounts (version 1.6.4) was used for gene counts, biotype counts, and rRNA estimation. Differential expression analysis was performed using the R package DEseq2 (version 1.26). Cutoff values of an absolute fold change of greater than 2 and an FDR of less than 0.1 were used to select for differentially expressed genes. GSEA was performed with the R package fgsea (version 1.14.0) using the “Hallmark” libraries from MsigDB.

#### ROR score.

We evaluated the PAM50 ROR score using RNA-Seq gene expression data (16, 17).

### CycIF

TMAs were constructed from 311 FFPE tumor blocks. After quality check, 174 samples (124 ED-S and 50 ED-R) from 118 patients were included in our analysis.

#### Preprocessing and prestaining of tissues for CycIF.

TMA sections (5 mm) were baked at 60°C for 1 hour, deparaffinized, rehydrated with serial passage through changes of xylene and graded alcohol, and washed in water. Multiplexed IF was performed using an adapted protocol from methodology described previously (39). TMA sections were processed as follows: the slides were deparaffinized; antigen retrieval was performed in a Cuisinart pressure cooker (model CPC-600) using pH 6 citrate buffer for 20 minutes, followed by a quick rinse in distilled water and incubation into pH 9 Tris/EDTA for 15 minutes. Slides were then blocked in a solution of PBS with 10% normal goat serum and 1% BSA. The autofluorescence level of each tissue was measured using an Axioscan fluorescence slide scanner (Zeiss). After this first round of autofluorescence (step 0), we proceeded to sequential staining, imaging, and quenching, in which each CycIF cycle involved 4 steps: (a) immunostaining with antibodies, (b) staining with DNA dye (DAPI) to mark nuclei and facilitate image registration, (c) image acquisition using 5 channels (DAPI, AF488, AF555, AF647, AF750), and (d) removal of the fluorescence signal through quenching and washing. After confirming the quenching of the IF signal, a new round of immunostaining started. The antibodies used are listed in Supplemental Table 1. A total of 9 rounds of CycIF were performed. Images were acquired using a fluorescence microscope (Axioscan, Zeiss). At the end of the process, the individual images were stitched together and subjected to image processing and segmentation to spatially identify individual cells. Coordinates (*x*, *y*) were extracted, together with cell phenotypes (based on nucleus size and roundness) and mean marker intensity for either the cytoplasm, the nucleus, or whole-cell antibody staining using QI Tissue software. Data were normalized and scaled using internal control tissues.

#### Cell subtyping and spatial analysis.

Cancer cells were identified and labeled using E-cadherin and/or pan-CK markers. To analyze cell-type enrichment in the TIME, we used proximal pressure analysis, whereby we estimated the overall effect caused by the expression of a protein (marker) in close proximity to each cancer cell (40). We defined the proximity constraint as the region within a 1-cell diameter around each cancer cell and defined the proximal pressure as the 90th percentile of expression of a protein in all cells within the proximity constraint. The cell diameter was estimated on the basis of the diameter of all cancer cells. Therefore, the proximal pressure data are expressed as *z* scores of marker expression. Median values from sensitive tumors were compared with those for resistant tumors.

### Spatial transcriptomics

#### Sample preparation and digital spatial profiler analysis.

FFPE tumor sections (5 μm) were mounted on charged slides, baked, and prepared on the Leica Biosystem, following the manufacturer’s protocol. After hybridization with RNA probes conjugated to barcoded oligonucleotide tags with a UV photocleavable linker and staining with fluorescent markers (pan-CK [epithelial cancer cells], CD45 [immune cells], SYTO13 [nucleus], and Ki67 [proliferation]), the slides were loaded onto a GeoMx digital spatial profiler (DSP) (NanoString Technologies) and scanned. We selected specific imaging ROIs of 222 × 354.6 μm, guided by an expert breast pathologist. Each ROI was further segmented into areas of illumination (AOIs) based on morphological features. The selected areas were then exposed to UV light, and barcoded oligonucleotides were released, aspirated, and dispensed into a collection plate for library construction for next-generation sequencing (NGS).

#### Library preparation and sequencing.

GeoMx NGS libraries were prepared following the manufacturer’s protocol. In brief, aspirates were dried at 65°C for 1 hour in a thermal cycler with an open lid and resuspended in 10 μL nuclease-free water. Rehydrated aspirates (4 μL) were mixed with 2 μL 5× PCR Master Mix and 4 μL SeqCode primers. PCR amplification was then performed for 18 cycles. The indexed libraries were pooled equally and purified twice with 1.2× AMPure XP beads (Beckman Coulter). The final libraries were evaluated and quantified using Agilent’s High Sensitivity DNA Kit and Invitrogen’s Qubit dsDNA HS assay, respectively. Total sequencing reads per DSP collection plate were calculated using the NanoString DSP Worksheet. Libraries were next sequenced using 38 bp paired-end sequencing (PE 38) on an Illumina NovaSeq 6000 system with a 100-cycle S1 kit (version 1.5).

#### Data processing and analysis.

Sequencing reads underwent trimming, merging, and alignment. Duplicates were removed with the reported count value for each gene within each sample representing the mean of individual probe counts after removal of outliers. The limit of quantification (LOQ) was determined as the geometric mean plus 2 SDs of the negative probes. Subsequently, FASTQ files were processed into digital count conversion files using NanoString’s GeoMx NGS Pipeline software. Quality control, data filtering, and normalization (Q3) were executed through the GeoMx DSP Data Analysis suite. Differential gene expression analysis was conducted using the R package limma (version 3.50.3), with *P* values adjusted by Benjamini-Hochberg correction. GSEA, investigating the human Molecular Signatures Database (MSigDB) Gene Ontology Biology Process gene sets, was carried out using GSEA software, version 4.3.2.

#### CIBERSORT.

Deconvolution of immune cells from bulk microarray expression data was performed using the CIBERSORT tool (22) with default parameters and the normalized protein-coding gene expression matrix as input. The significance of changes in immune cell estimates for unpaired samples was calculated using the Wilcoxon rank-sum test.

### Cell lines and reagents

MCF-7 (ATCC HTB-22), T47D (HTB-133), HCC-1428 (CRL-2327), and ZR75-1 (CRL-1500) human breast cancer cell lines were obtained from the American Type Culture Collection (ATCC) and maintained in ATCC-recommended media supplemented with 10% FBS (Gibco, Thermo Fisher Scientific) and 1× antibiotic/antimycotic (Gibco, Thermo Fisher Scientific) at 37°C in a humidified atmosphere of 5% CO_2_. TALL-104 (CRL-11386), a human CD8^+^ T cell acute leukemia cell line, was obtained from ATCC and maintained in RPMI supplemented with 10% heat-inactivated FBS and 1× antibiotic/antimycotic (Gibco, Thermo Fisher Scientific) plus IL-2 improved sequence (Miltenyi Biotec, 130097744), at 37°C in a humidified atmosphere of 5% CO_2_. Cell lines were authenticated by ATCC by the short tandem repeat (STR) method. Mycoplasma testing was conducted for each cell line before use and every 6 months (or less) thereafter using the MycoAlert Mycoplasma Detection Kit (Lonza, catalog LT07-710). In ED experiments, the cell lines were seeded in phenol red–free RPMI plus 10% charcoal-stripped FBS plus 1× antibiotic/antimycotic (Gibco, Thermo Fisher Scientific). Recombinant CXCL9, CXCL10, and CXCL11 were purchased from R&D System (catalog 392-MG-050, catalog 266-IP-050, catalog 672-IT-025, respectively). IncuCyte Nuclight Rapid Red Dye (catalog 4717) was purchased from Sartorius and used for nuclear labeling of live cells in the experiments performed with the IncuCyte Live-Cell Analysis System.

### Immunoblot analysis

Cells were lysed with RIPA lysis buffer supplemented with protease inhibitors (Mini, EDTA-free Protease Inhibitor Cocktail, Roche) and phosphatase inhibitor (PhosSTOP, Roche). Protein concentration was determined using Pierce BCA Protein Assay Reagents (Thermo Fisher Scientific). Proteins were separated by 4%–12% NuPAGE gradient gels (Invitrogen, Thermo Fisher Scientific), transferred onto nitrocellulose membranes, blocked with 5% milk at room temperature for 1 hour, and then probed with primary antibodies at 4°C overnight. Incubation with HRP-conjugated anti-rabbit antibodies, as a secondary antibody, was performed for 1 hour at room temperature. Protein bands were detected with an ECL substrate (Pierce ECL Western Blotting substrate, Thermo Fisher Scientific) using the ChemiDoc Imaging System (Bio-Rad). The following primary antibodies were used: rabbit anti-vinculin (Cell Signaling Technology, catalog 13901), rabbit anti-pERK1/2 (Cell Signaling Technology, catalog 9101), rabbit anti-ERK1/2 (Cell Signaling Technology, catalog 9102), rabbit anti-AKT1 (S473) (Cell Signaling Technology, catalog 4060), rabbit anti-AKT1 (Cell Signaling Technology, catalog 75692), rabbit anti-CXCR3 (Abcam, catalog ab288437), and rabbit anti-CXCR7 (GeneTex, catalog GTX100027).

### qRT-PCR

Total RNA was reverse transcribed to cDNA using the iScript kit (Bio-Rad). qRT-PCR was performed using SYBR Green Master Mix (Thermo Fisher Scientific) on a QuantStudio 3 Real-Time PCR System (Thermo Fisher Scientific). Expression of *YWHAZ* was used as an internal control for normalization. The primers used were as follows: *YWHAZ* (Qiagen, catalog 330001 PPH01017A), *CXCR3* (Qiagen, catalog 330001 PPH01041A), and *CXCR7* (Qiagen, catalog 330001 PPH01182F).

### Generation of knockout cell lines

LentiCRISPR-v2 plasmids containing the following sgRNAs were purchased from GenScript: constructs targeting CXCR3 (IDs: HP044932 and HP044935) and CXCR7 (IDs: HP044929 and HP044916). For lentivirus production, HEK293FT cells were cotransfected with sgRNA-containing plasmids and packaging plasmids (psPAX2 and pMD2.G). After 24 hours, the culture medium was replaced with fresh medium, and viral supernatants were collected twice, 24 hours and 48 hours later. MCF7 cells were transduced with the viral supernatants in the presence of polybrene (8 μg/mL) and subsequently selected with puromycin (1 μg/mL).

### siRNA transfection

Silencer select siRNAs targeting CXCR3 (ID: s6013/), CXCR7 (ID: s94), and a nontargeting control (catalog 4390843) were purchased from Invitrogen (Thermo Fisher Scientific). Cells were transfected with 30 nM siRNA-3, 20 nM siRNA-7, or the corresponding concentration-matched control siRNAs (siRNA-CTRL) using Lipofectamine RNAiMAX (Thermo Fisher Scientific) following the manufacturer’s instructions. Forty-eight hours after transfection, cells were seeded in 24-well plates with an additional 10 nM siRNA in EFM. After 24 hours, either T-ALL-104 cocultures or CXCL11 were added.

### ELISA

Quantification of CXCL9, CXCL10, and CXCL11 in the conditioned media of the cocultures was performed using the Human CXCL9/MIG Quantikine ELISA Kit (catalog DCX900), the Human CXCL10/IP-10 Quantikine ELISA Kit (catalog DIP100), and the Human CXCL11/I-TAC Quantikine ELISA Kit (catalog DCX110), respectively, according to the manufacturer’s instructions (R&D Systems).

### Outcome analysis

The KM Plotter online tool is a public database (http://www.kmplot.com) containing information on more than 35,000 samples from 21 tumor types, including breast cancer. This tool was applied to analyze the correlation between CXCL9, CXCL10, CXCL11 expression and RFS among patients with HR^+^ HER2^–^ breast cancer who underwent endocrine therapy. The Kaplan-Meier method was used to plot RFS curves, and the log-rank test was used for comparisons. The ROC curve was used to evaluate the predictive value of *CXCL11* gene expression in the cohort. The AUC was calculated to evaluate the performance of the model.

### Statistics

Statistical analysis was performed using GraphPad Prism, version 10.1.2 (GraphPad Software). For comparisons between 2 groups, unpaired, 2-tailed *t* tests or 2-tailed Mann-Whitney *U* tests were performed, whereas in the case of multiple comparisons, a Kruskal-Wallis nonparametric test was applied. *P* value correction for multiple comparison was obtained by controlling the FDR. A *P* value of less than 0.05 was considered statistically significant. For RNA-Seq analysis and GSEA, a FDR below 0.05 was considered statistically significant. Data represent the mean ± SD.

### Study approval

FFPE blocks from primary breast tumor samples were obtained from the patients enrolled in the trial NCT00651976. The study was approved by the IRBs of Vanderbilt University Medical Center and UT Southwestern Medical Center. All patients provided written informed consent. Medical reports were obtained without personally identifiable information.

### Data availability

Raw data from RNA-Seq and spatial transcriptomics have been deposited in the NCBI’s Gene Expression Omnibus (GEO) database (accession numbers GSE297605 and GSE299880, respectively). Processed image data are available on figshare (https://figshare.com/articles/dataset/normalized_cycif_data/25944580). Values for all data points in graphs are reported in the Supporting Data Values file.

## Author contributions

FN, ABH, and CLA conceptualized the study. FN, YW, and DRS analyzed data. HM, NE, GBM, and ML performed CycIF. YW analyzed CycIF data. LG and JZ performed spatial transcriptomics analysis. PIGE and LF performed tissue processing. PIGE and YVF performed tissue analysis. NU, SS, JMB, and CLA enrolled patients in the trial. YW, JZ, LG, KA, SB, LX, JL, and TW performed bioinformatics analysis. MRCP, CCL, YU, KML, AS, SM, YM, and PL performed additional analysis and experiments. FN wrote the manuscript. AHB, RB, GBM, and CLA provided guidance. FN, ABH, and CLA edited the manuscript. CCL, ABH, and CLA provided funding.

## Funding support

This work is the result of NIH funding, in whole or in part, and is subject to the NIH Public Access Policy. Through acceptance of this federal funding, the NIH has been given a right to make the work publicly available in PubMed Central

- National Cancer Institute (NCI), NIH (P30 CA142543, to CLA).
- Department of Defense (DOD) (BC 210406, to CCL).
- NCI, NIH (R01CA273246, to CLA and ABH).
- NCI, NIH Breast SPORE (P50 CA098131, to CLA and ABH).
- NCI (P30 CA1142543, to the Simmons Comprehensive Cancer Center).
- Cancer Prevention and Research Institute of Texas (CPRIT) (RR170061, to CLA).
- Susan G. Komen Breast Cancer Foundation (SAB1800010, to CLA).
- Breast Cancer Research Foundation (to CLA and ABH).

## Supplementary Material

Supplemental data

Unedited blot and gel images

Supporting data values

## Figures and Tables

**Table 1:**
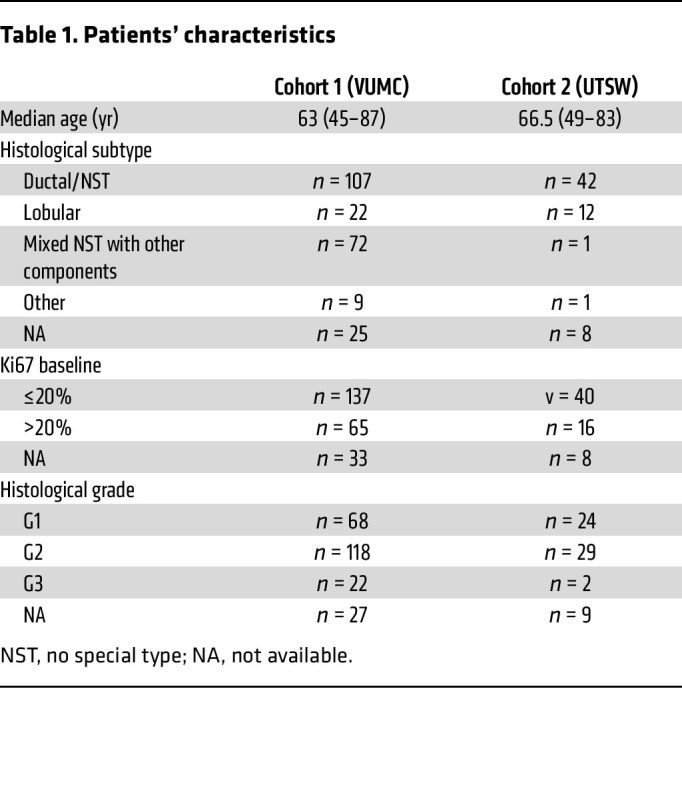
Patients’ characteristics

## Version 1 12/09/2025

In-Press Preview

## Version 2 02/02/2026

Electronic publication

## References

[1] Harbeck N (2019). Breast cancer. Nat Rev Dis Primers.

[2] Hanker AB (2020). Overcoming endocrine resistance in breast cancer. Cancer Cell.

[3] García-Teijido P (2016). Tumor-infiltrating lymphocytes in triple negative breast cancer: the future of immune targeting. Clin Med Insights Oncol.

[4] Luen SJ (2017). Tumour-infiltrating lymphocytes in advanced HER2-positive breast cancer treated with pertuzumab or placebo in addition to trastuzumab and docetaxel: a retrospective analysis of the CLEOPATRA study. Lancet Oncol.

[5] Stanton SE (2016). Variation in the incidence and magnitude of tumor-infiltrating lymphocytes in breast cancer subtypes: a systematic review. JAMA Oncol.

[6] Loi S (2013). Prognostic and predictive value of tumor-infiltrating lymphocytes in a phase III randomized adjuvant breast cancer trial in node-positive breast cancer comparing the addition of docetaxel to doxorubicin with doxorubicin-based chemotherapy: BIG 02-98. J Clin Oncol.

[7] Denkert C (2018). Tumour-infiltrating lymphocytes and prognosis in different subtypes of breast cancer: a pooled analysis of 3771 patients treated with neoadjuvant therapy. Lancet Oncol.

[8] Sobral-Leite M (2019). Cancer-immune interactions in ER-positive breast cancers: PI3K pathway alterations and tumor-infiltrating lymphocytes. Breast Cancer Res.

[9] Dunbier AK (2013). Molecular profiling of aromatase inhibitor-treated postmenopausal breast tumors identifies immune-related correlates of resistance. Clin Cancer Res.

[10] Skriver SK (2020). Tumour-infiltrating lymphocytes and response to neoadjuvant letrozole in patients with early oestrogen receptor-positive breast cancer: analysis from a nationwide phase II DBCG trial. Breast Cancer Res.

[11] Schuster EF (2023). Molecular profiling of aromatase inhibitor sensitive and resistant ER+HER2- postmenopausal breast cancers. Nat Commun.

[12] Hazlett J (2021). Oestrogen deprivation induces chemokine production and immune cell recruitment in in vitro and in vivo models of oestrogen receptor-positive breast cancer. Breast Cancer Res.

[13] Siersbæk R (2020). IL6/STAT3 signaling hijacks estrogen receptor α enhancers to drive breast cancer metastasis. Cancer Cell.

[14] Smith IE (2005). Neoadjuvant treatment of postmenopausal breast cancer with anastrozole, tamoxifen, or both in combination: the Immediate Preoperative Anastrozole, Tamoxifen, or Combined with Tamoxifen (IMPACT) multicenter double-blind randomized trial. J Clin Oncol.

[15] Osborne CK, Schiff R (2011). Mechanisms of endocrine resistance in breast cancer. Annu Rev Med.

[16] Fernandez-Martinez A (2020). Survival, pathologic response, and genomics in CALGB 40601 (Alliance), a neoadjuvant phase III trial of paclitaxel-trastuzumab with or without lapatinib in HER2-positive breast cancer. J Clin Oncol.

[17] Parker JS (2009). Supervised risk predictor of breast cancer based on intrinsic subtypes. J Clin Oncol.

[18] Salgado R (2015). The evaluation of tumor-infiltrating lymphocytes (TILs) in breast cancer: recommendations by an International TILs Working Group 2014. Ann Oncol.

[19] Lin JR (2016). Cyclic immunofluorescence (CycIF), a highly multiplexed method for single-cell imaging. Curr Protoc Chem Biol.

[20] van der Leun AM (2020). CD8^+^ T cell states in human cancer: insights from single-cell analysis. Nat Rev Cancer.

[21] Newman AM (2015). Robust enumeration of cell subsets from tissue expression profiles. Nat Methods.

[22] Tokunaga R (2018). CXCL9, CXCL10, CXCL11/CXCR3 axis for immune activation - a target for novel cancer therapy. Cancer Treat Rev.

[23] Dowsett M (2005). Short-term changes in Ki-67 during neoadjuvant treatment of primary breast cancer with anastrozole or tamoxifen alone or combined correlate with recurrence-free survival. Clin Cancer Res.

[24] Onkar S (2023). Immune landscape in invasive ductal and lobular breast cancer reveals a divergent macrophage-driven microenvironment. Nat Cancer.

[25] Tietscher S (2023). A comprehensive single-cell map of T cell exhaustion-associated immune environments in human breast cancer. Nat Commun.

[26] Weigelin B, Friedl P (2022). T cell-mediated additive cytotoxicity - death by multiple bullets. Trends Cancer.

[27] Dorner BG (2009). Selective expression of the chemokine receptor XCR1 on cross-presenting dendritic cells determines cooperation with CD8^+^ T cells. Immunity.

[28] Nanda R (2020). Effect of pembrolizumab plus neoadjuvant chemotherapy on pathologic complete response in women with early-stage breast cancer: an analysis of the ongoing phase 2 adaptively randomized I-SPY2 trial. JAMA Oncol.

[29] Loi S (2023). LBA20 A randomized, double-blind trial of nivolumab (NIVO) versus placebo (PBO) with neoadjuvant chemotherapy (NACT) followed by adjuvant endocrine therapy (ET) ± NIVO in patients (pts) with high-risk, ER^+^ HER2^−^ primary breast cancer (BC). Ann Oncol.

[30] Cardoso F (2023). LBA21 KEYNOTE-756: Phase III study of neoadjuvant pembrolizumab (pembro) or placebo (pbo) + chemotherapy (chemo), followed by adjuvant pembro or pbo + endocrine therapy (ET) for early-stage high-risk ER^+^/HER2^–^ breast cancer. Ann Oncol.

[31] Cobain EF (2024). Elucidating the immune active state of HR^+^HER2^–^ MammaPrint High 2 early breast cancer. J Clin Oncol.

[32] Giltnane JM (2017). Genomic profiling of ER^+^ breast cancers after short-term estrogen suppression reveals alterations associated with endocrine resistance. Sci Transl Med.

[33] Formisano L (2017). Association of FGFR1 with ERα maintains ligand-independent ER transcription and mediates resistance to estrogen deprivation in ER^+^ breast cancer. Clin Cancer Res.

[34] Guerrero-Zotano AL (2018). ER^+^ breast cancers resistant to prolonged neoadjuvant letrozole exhibit an E2F4 transcriptional program sensitive to CDK4/6 inhibitors. Clin Cancer Res.

[35] Servetto A (2021). Nuclear FGFR1 regulates gene transcription and promotes antiestrogen resistance in ER^+^ breast cancer. Clin Cancer Res.

[36] Brown JR (2014). Quantitative assessment Ki-67 score for prediction of response to neoadjuvant chemotherapy in breast cancer. Lab Invest.

[37] Jeselsohn R (2014). Emergence of constitutively active estrogen receptor-α mutations in pretreated advanced estrogen receptor-positive breast cancer. Clin Cancer Res.

[38] Chung GG (2007). Quantitative analysis of estrogen receptor heterogeneity in breast cancer. Lab Invest.

[39] Labrie M (2021). Multiomics analysis of serial PARP inhibitor treated metastatic TNBC inform on rational combination therapies. NPJ Precis Oncol.

[40] Wang Y (2022). Sprod for de-noising spatially resolved transcriptomics data based on position and image information. Nat Methods.

