# Supplementary material for: CD8+ T cells in the tumor microenvironment modulate the response to endocrine therapy in breast cancer
Source: J Clin Invest. 2025 Dec 9;136(3):e188458. doi: 10.1172/JCI188458 (PMC12867153; doi:10.1172/JCI188458)
Supplement: Supplemental data [file jci-136-188458-s275.pdf]

Supplemental Data for

CD8+ T cells in the tumor microenvironment modulate response to endocrine therapy in breast cancer

Authors:

Fabiana Napolitano[1,2], Yunguan Wang[3,4], Dhivya R. Sudhan[1†], Paula I. González-Ericsson[5], Luigi Formisano[2], Nisha Unni[6], Shahbano Shakeel[6], James Zhu[7], Khushi Ahuja[8], Lei Guo[7], María Rosario Chica-Parrado[1], Yuki Matsunaga[1], Pamela Luna[1], Chang-Ching Lin[1], Yasuaki Uemoto[1], Kyung-Min Lee[1,9], Hongli Ma[10], Nathaniel Evans[10,11], Alberto Servetto[1,2], Saurabh Mendiratta[1], Spencer Barnes[8], Roberto Bianco[2], Yisheng V. Fang[12], Lin Xu[7], Jeon Lee[8], Tao Wang[7], Justin M. Balko[5,13], Gordon B. Mills[14], Marilyne Labrie[15,16,17], Ariella B. Hanker[1*], Carlos L. Arteaga[1*]

1. Harold C. Simmons Comprehensive Cancer Center, University of Texas Southwestern Medical Center, Dallas, TX, USA.
2. Department of Clinical Medicine and Surgery, University of Naples Federico II, Naples, Italy.
3. Division of Pediatric Gastroenterology, Hepatology and Nutrition. Cincinnati Children's Hospital Medical Center, Cincinnati, OH, USA
4. Department of Pediatrics, University of Cincinnati, Cincinnati, OH, USA
5. Departments of Medicine, Vanderbilt-Ingram Cancer Center, Vanderbilt University Medical Center, Nashville, TN, USA.
6. Department of Medical Oncology, University of Texas Southwestern Medical Center, Dallas, TX, USA
7. Quantitative Biomedical Research Center, Peter O'Donnell Jr. School of Public Health, University of Texas Southwestern Medical Center, Dallas, TX, USA.
8. Lyda Hill Department of Bioinformatics, University of Texas Southwestern Medical Center, Dallas, TX, USA.
9. Department of Life Sciences, College of Natural Science, Hanyang University, Seoul, Republic of South Korea.
10. Knight Cancer Institute, Oregon Health & Science University, Portland, OR, United States.
11. Division of Bioinformatics & Computational Biology, Department of Medical Informatics and Clinical Epidemiology, Oregon Health & Science University, Portland, OR, USA
12. Department of Pathology, UT Southwestern Medical Center, Dallas, TX, USA.
13. Department of Pathology, Microbiology & Immunology, Vanderbilt-Ingram Cancer Center, Vanderbilt University Medical Center, Nashville, TN, USA.
14. Division of Oncological Sciences, Knight Cancer Institute, Oregon Health & Science University, Portland, OR, USA.
15. Department of Immunology and Cell Biology, Faculty of medicine and health science, Université de Sherbrooke, Canada.

16. Centre de Recherche du Centre Hospitalier de l'Université de Sherbrooke, Canada.
17. Institut de Recherche sur le Cancer de l'Université de Sherbrooke, Canada.
† Current affiliation: Oncology R&D, AstraZeneca, Waltham, MA 02451, USA.

Corresponding authors:
Carlos L. Arteaga, MD
H. Simmons Comprehensive Cancer Center, University of Texas Southwestern Medical Center, 5323 Harry Hines Blvd., Dallas, TX 75390
Phone: +1 214-648-4190. Email: Carlos.Arteaga@UTSouthwestern.edu;

Ariella B. Hanker, PhD
H. Simmons Comprehensive Cancer Center, University of Texas Southwestern Medical Center, 5323 Harry Hines Blvd., Dallas, TX 75390
Phone: +1 214-648-8344. Email: ariella.hanker@UTSouthwestern.edu.

Supplemental Table 1. List of CycIF antibodies.

Supplemental Table 2 (xlsx)**.** Pathways significantly upregulated in PanCK+ cells in onTx (vs. preTx) ED-sensitive tumors.

Supplemental Table 3 (xlsx)**.** Pathways significantly upregulated in CD45+ cells in onTx (vs. preTx) ED-sensitive tumors.

Supplemental Table 4 (xlsx)**.** Pathways significantly upregulated in PanCK+ cells in preTx ED-sensitive (vs pre-Tx ED-resistant).

Supplemental Table 5 (xlsx)**.** Pathways significantly upregulated in CD45+ cells in preTx ED-sensitive (vs pre-Tx ED-resistant).

Supplemental Table 6 (xlsx)**.** Pathways significantly upregulated in PanCK+ cells in preTx ED-resistant (vs pre-Tx ED-sensitive).

Supplemental Table 7 (xlsx)**.** Pathways significantly upregulated in CD45+ cells in preTx ED-resistant (vs pre-Tx ED-sensitive).

Supplemental Figures 1-11.

Supplemental Table 1. List of CycIF antibodies.

Name	Company	Cat. No	Product Name
53BP1	Abcam	ab222232	Anti-53BP1 antibody [EPR2172(2)] (ab175933)
AR	CST	8956	Androgen Receptor (D6F11) XP® Rabbit mAb (Alexa Fluor® 555 Conjugate) #
AXL	R&D	AF154	Human Axl Antibody
B7-H4	Abcam	ab222927	Anti-B7H4 antibody [EPR20236] - Low endotoxin, Azide free
Bcl-xL	Abcam	ab200527	Recombinant Anti-Bcl-XL antibody [EPR16642] (Alexa Fluor® 488) (ab200617)
BRCA1	Abcam	ab215988	Anti-BRCA1 antibody [EPR19433] - BSA and Azide free
CCNB1	Abcam	ab214381	Anti-Cyclin B1 antibody [Y106] (Alexa Fluor® 555)
CCND1	Abcam	Ab190563	Anti-Cyclin D1 antibody [EPR2241] (Alexa Fluor® 647)
CCNE	lifetech	50-9714-80	Cyclin E Antibody, eFluor® 660
CD4	abcam	ab196147	Anti-CD4 antibody [EPR6855] (Alexa Fluor 647) (ab196147)
CD44	Novus	NBP1-47386AF750	CD44 Antibody (8E2F3) [Alexa Fluor® 750]
CD45	Novus	NB100-77417	CD45 [30F11] Alexa-Fluor750 antibody
CD68	Biolegend	916104	Purified anti-CD68 Antibody (KP1)
CD8	Invitrogen	50-0008-82	Anti-Hu CD8a [AMC908] eFluor660
CK19*	Biolegend	628502	Purified anti-Cytokeratin 19 Antibody
CK5*	Biolegend	905501	Keratin 5 Polyclonal Antibody, Purified (1 mg/ml) 0.03% Thimerosal
CK7*	abcam	ab185048	Anti-Cytokeratin 7 antibody [EPR1619Y] - Cytoskeleton Marker (Alexa Fluor 488) (ab185048)
CK8*	abcam	ab192467	Anti-Cytokeratin 8 antibody [EP1628Y] (Alexa Fluor 488) (ab192467)
E-Cadherin	Novus	NBP2-54587AF750	E-Cadherin Antibody (4A2) [Alexa Fluor® 750]
ERa	Abcam	ab205851	Anti-Estrogen Receptor alpha antibody [EPR4097] (Alexa Fluor 647) (ab205851)

Fibronectin	Abcam	ab32419	Anti-Fibronectin antibody [F1] (ab32419)
FOXP3	Biolegend	320102	Purified anti-human FOXP3 Antibody (206D)
H3K27Me3	CST	5499	Tri-Methyl-Histone H3 (Lys27) (C36B11) Rabbit mAb (Alexa Fluor 488 Conjugate) #5499
HER2	Thermo	MA5-13675	ErbB2 (HER-2) Monoclonal Antibody (3B5), PBS, pH 7.4, with 0.2% BSA
ki67	Novus	NB110-90592AF750	Ki67/MKI67 Antibody [Alexa Fluor® 750]
pATM	Abcam	ab81292	Anti-ATM (phospho S1981) antibody [EP1890Y]
pATR	Abcam	ab230831	Anti-ATR (phospho S428) antibody [EPR2184] - BSA and Azide free (ab230831)
PCNA	CST	8580	PCNA (PC10) Mouse mAb (Alexa Fluor 488 Conjugate) #8580
PD1	abcam	ab201825	Anti-PD1 antibody [EPR4877(2)] (Alexa Fluor 647) (ab201825)
pERK	R&D	AF1018	Human/Mouse/Rat Phospho-ERK1(T202/Y204)/ERK2 (T185/Y187) Antibody
pRB	abcam	ab215947	Anti-Rb (phospho S807) antibody [EPR17732] (Alexa Fluor® 647) (ab215947)
pS6	CST	3985S	Phospho-S6 Ribosomal Protein (Ser235/236) (D57.2.2E) XP Rabbit mAb (Alexa Fluor 555 Conjugate) #3985
PTEN	R&D	AF847	Human/Mouse/Rat PTEN Antibody
Sting	Abcam	ab198952	Anti-TMEM173 antibody [EPR13130] (Alexa Fluor® 647)
TP53	Bioss	bs-8687R-A488	p53 (FL-393) Polyclonal Antibody, ALEXA FLUOR® 488 Conjugated
Vimentin	CST	9854	Vimentin (D21H3) XP Rabbit mAb (Alexa Fluor 488 Conjugate) #9854
CD20	Abcam	ab78237	Anti-CD20 antibody [EP459Y]
pHH3	CST	3465S	Phospho-Histone H3 (Ser10) (D2C8) XP Rabbit mAb (Alexa Fluor 488 Conjugate) #3465

* CKS = mix of 4 anti-cytokeratins antibody

Supplementary Table 2. List of pathways enriched in PanCK+ cells in onTx (vs. preTx) ED-sensitive tumors.

NAME	NES	NOM p-val	FDR q-val
GOBP_COLLAGEN_FIBRIL_ORGANIZATION	-2.5509026	0	0.00018048
GOBP_RESPONSE_TO_MUSCLE_STRETCH	-2.3517835	0	0.00448775
GOBP_CYTOPLASMIC_TRANSLATION	-2.300575	0	0.00669516
GOBP_REGULATION_OF_P38MAPK_CASCADE	-2.229678	0	0.01450056
GOBP_RESPONSE_TO_EPIDERMAL_GROWTH_FACTOR	-2.1846328	0	0.02145791
GOBP_CELLULAR_RESPONSE_TO_COPPER_ION	-2.120218	0	0.03857531
GOBP_FACE_MORPHOGENESIS	-2.1068316	0.00045025	0.03944699

Supplementary Table 3. List of pathways enriched in CD45+ cells in onTx (vs. preTx) ED-sensitive tumors.

**NAME**	**NES**	**NOM p-val**	**FDR q-val**
GOBP_DENDRITIC_CELL_CHEMOTAXIS	-2.05855	0	0.000155665
GOBP_POSITIVE_T_CELL_SELECTION	-1.98834	0	0.00102052
GOBP_RESPONSE_TO_CHEMOKINE	-1.94921	0	0.002575143
GOBP_DENDRITIC_CELL_MIGRATION	-1.921	0	0.004106511
GOBP_NEGATIVE_REGULATION_OF_TOLL_LIKE_RECEPTOR_SIGNALING_PATHWAY	-1.9187	0	0.004158817
GOBP_B_CELL_RECEPTOR_SIGNALING_PATHWAY	-1.90168	0	0.005070204
GOBP_SPERM_FLAGELLUM_ASSEMBLY	-1.89657	0	0.005444758
GOBP_T_CELL_SELECTION	-1.8908	0	0.005745839
GOBP_ESTABLISHMENT_OF_LYMPHOCYTE_POLARITY	-1.86628	0.001217	0.008697821
GOBP_SENSORY_PERCEPTION_OF_SMELL	-1.83879	0	0.01377314
GOBP_SLEEP	-1.83503	0	0.014222567
GOBP_REGULATION_OF_T_HELPER_1_TYPE_IMMUNE_RESPONSE	-1.82991	0	0.015108234
GOBP_NEUROPEPTIDE_SIGNALING_PATHWAY	-1.82487	0	0.016117137
GOBP_TOLERANCE_INDUCTION	-1.82166	0	0.016534299
GOBP_REGULATION_OF_CELL_KILLING	-1.79495	0	0.024503766
GOBP_DETECTION_OF_STIMULUS_INVOLVED_IN_SENSORY_PERCEPTION	-1.79321	0	0.024657255
GOBP_POSITIVE_REGULATION_OF_RECEPTOR_SIGNALING_PATHWAY_VIA_STAT	-1.79279	0	0.024359372
GOBP_KERATINIZATION	-1.78936	0	0.02524492
GOBP_ANTIGEN_RECEPTOR_MEDIATED_SIGNALING_PATHWAY	-1.78604	0	0.02594164
GOBP_DENDRITIC_CELL_CHEMOTAXIS	-1.78023	0	0.027955929
GOBP_REGULATION_OF_CILIUM_DEPENDENT_CELL_MOTILITY	-1.7708	0	0.03176934
GOBP_PURINERGIC_NUCLEOTIDE_RECEPTOR_SIGNALING_PATHWAY	-1.76761	0.001164	0.032782726
GOBP_SPERM_AXONEME_ASSEMBLY	-1.767	0	0.03228566
GOBP_T_CELL_CHEMOTAXIS	-1.76555	0	0.03225527
GOBP_ALPHA_BETA_T_CELL_ACTIVATION	-1.75763	0	0.035661146
GOBP_POSITIVE_REGULATION_OF_CELL_KILLING	-1.75537	0	0.03604166
GOBP_NEGATIVE_REGULATION_OF_OXIDOREDUCTASE_ACTIVITY	-1.75387	0	0.035863217
GOBP_B_CELL_PROLIFERATION	-1.75298	0.00227	0.035583213

GOBP_POSITIVE_REGULATION_OF_ALPHA_BETA_T_CELL_PROLIFERATION	-1.7512	0	0.036004797
GOBP_HETEROPHILIC_CELL_CELL_ADHESION_VIA_PLASMA_MEMBRANE_CELL_ADHESION_MOLECULES	-1.74467	0	0.039048597
GOBP_SPERM_MOTILITY	-1.7404	0	0.04057833
GOBP_FEEDING_BEHAVIOR	-1.73459	0	0.04341755
GOBP_LYMPHOCYTE_CHEMOTAXIS	-1.73282	0.001145	0.04373024
GOBP_REGULATION_OF_T_CELL_DIFFERENTIATION_IN_THYMUS	-1.73148	0.002339	0.044046693
GOBP_POSITIVE_REGULATION_OF_NATURAL_KILLER_CELL_MEDIATED_CYTOTOXICITY	-1.73123	0.001148	0.04340693
GOBP_REGULATION_OF_SYNAPTIC_TRANSMISSION_GABAERGIC	-1.72997	0	0.043572094
GOBP_CILIUM_MOVEMENT	-1.72688	0.004745	0.04435881
GOBP_GAMMA_DELTA_T_CELL_ACTIVATION	-1.72608	0.003589	0.04414328
GOBP_IMMUNOLOGICAL_SYNAPSE_FORMATION	-1.72587	0	0.043605365
GOBP_NEGATIVE_REGULATION_OF_T_CELL_MEDIATED_IMMUNITY	-1.72487	0	0.04355697
GOBP_T_CELL_RECEPTOR_SIGNALING_PATHWAY	-1.72265	0	0.044086732
GOBP_CILIUM_OR_FLAGELLUM_DEPENDENT_CELL_MOTILITY	-1.72229	0.0047	0.043658182
GOBP_DENDRITE_EXTENSION	-1.71653	0.003308	0.046386693
GOBP_VITAMIN_TRANSPORT	-1.71222	0	0.048462436
GOBP_ANTIMICROBIAL_HUMORAL_RESPONSE	-1.70983	0	0.049286176
GOBP_REGULATION_OF_HUMORAL_IMMUNE_RESPONSE	-1.70923	0.002301	0.049048357
GOBP_REGULATION_OF_TOLERANCE_INDUCTION	-1.70602	0	0.049724933
GOBP_REGULATION_OF_NATURAL_KILLER_CELL_MEDIATED_IMMUNITY	-2.05855	0	0.000155665

Supplementary Table 4. Pathways upregulated in PanCK+ cells in preTx ED-sensitive (vs

NAME	NES	NOM p-val	FDR q-val
GOBP_MALE_MEIOSIS_I	2.124276	0	0.0124424

Supplementary Table 5. Pathways upregulated in CD45+ cells in preTx ED-sensitive (vs pre-Tx ED-resistant)

NAME	NES	NOM p-val	FDR q-val
GOBP_PROTON_MOTIVE_FORCE_DRIVEN_ATP_SYNTHESIS	2.394424	0	0
GOBP_ATP_BIOSYNTHETIC_PROCESS	2.231166	0	0.02374952
GOBP_EPITHELIAL_TUBE_BRANCHING_INVOLVED_IN_LUNG_MORPHOGENESIS	2.209022	0	0.02336747

Supplementary Table 6. Pathways significantly upregulated in PanCK+ cells in preTx ED-resistant (vs pre-Tx ED-sensitive)

NAME	NES	NOM p-val	FDR q-val
GOBP_RESPONSE_TO_HYPEROXIA	-2.223915	0	0.02554317
GOBP_MAMMARY_GLAND_EPITHELIAL_CELL_DIFFERENTIATION	-2.191237	0	0.02550599
GOBP_HIPPO_SIGNALING	-2.145774	0	0.02699748

Supplementary Table 7. Pathways significantly upregulated in CD45+ cells in preTx ED-resistant (vs pre-Tx ED-sensitive)

NAME	NES	NOM p-val	FDR q-val
GOBP_ALPHA_BETA_T_CELL_ACTIVATION	-2.2766364	0	0
GOBP_POSITIVE_REGULATION_OF_INTERLEUKIN_2_PRODUCTION	-2.2229671	0	0
GOBP_POSITIVE_REGULATION_OF_T_CELL_PROLIFERATION	-2.2213304	0	0
GOBP_ANTIGEN_RECEPTOR_MEDIATED_SIGNALING_PATHWAY	-2.2113037	0	0
GOBP_T_CELL_RECEPTOR_SIGNALING_PATHWAY	-2.2085752	0	0
GOBP_ADAPTIVE_IMMUNE_RESPONSE	-2.1861205	0	0
GOBP_POSITIVE_T_CELL_SELECTION	-2.176356	0	0
GOBP_T_CELL_ACTIVATION	-2.1718414	0	0
GOBP_LYMPHOCYTE_COSTIMULATION	-2.164869	0	9.27E-05
GOBP_POSITIVE_REGULATION_OF_CD4_POSITIVE_ALPHA_BETA_T_CELL_ACTIVATION	-2.1621838	0	8.34E-05
GOBP_REGULATION_OF_ALPHA_BETA_T_CELL_ACTIVATION	-2.1477053	0	1.51E-04
GOBP_POSITIVE_REGULATION_OF_ALPHA_BETA_T_CELL_ACTIVATION	-2.1330566	0	1.39E-04
GOBP_ANTIGEN_PROCESSING_AND_PRESENTATION_VIA_MHC_CLASS_IB	-2.130435	0	1.28E-04
GOBP_T_CELL_PROLIFERATION	-2.0883007	0	3.56E-04
GOBP_POSITIVE_REGULATION_OF_CELL_KILLING	-2.0878506	0	3.32E-04
GOBP_COLLAGEN_FIBRIL_ORGANIZATION	-2.07671	0	5.20E-04
GOBP_ALPHA_BETA_T_CELL_PROLIFERATION	-2.0681708	0	6.86E-04
GOBP_CELLULAR_DEFENSE_RESPONSE	-2.062868	0	7.86E-04
GOBP_IMMUNOLOGICAL_SYNAPSE_FORMATION	-2.0625434	0	7.45E-04

GOBP_POSITIVE_REGULATION_OF_LEUKOCYTE_PROLIFERATION	-2.061587	0	7.08E-04
GOBP_CD4_POSITIVE_ALPHA_BETA_T_CELL_ACTIVATION	-2.059414	0	7.92E-04
GOBP_REGULATION_OF_T_CELL_ACTIVATION	-2.0576794	0	7.56E-04
GOBP_POSITIVE_REGULATION_OF_ALPHA_BETA_T_CELL_PROLIFERATION	-2.0441139	0	0.001085282
GOBP_ANTIGEN_PROCESSING_AND_PRESENTATION_OF_ENDOGENOUS_ANTIGEN	-2.0428052	0	0.001074968
GOBP_REGULATION_OF_LEUKOCYTE_PROLIFERATION	-2.0394578	0	0.001133305
GOBP_REGULATION_OF_CD4_POSITIVE_ALPHA_BETA_T_CELL_ACTIVATION	-2.038939	0	0.001089716
GOBP_CD4_POSITIVE_ALPHA_BETA_T_CELL_DIFFERENTIATION	-2.0304594	0	0.001296857
GOBP_CELL_KILLING	-2.0286582	0	0.001310241
GOBP_ALPHA_BETA_T_CELL_DIFFERENTIATION	-2.0261638	0	0.001322127
GOBP_T_HELPER_17_TYPE_IMMUNE_RESPONSE	-2.0204892	0	0.001416778
GOBP_REGULATION_OF_LYMPHOCYTE_ACTIVATION	-2.004998	0	0.001936008
GOBP_POSITIVE_REGULATION_OF_CD4_POSITIVE_ALPHA_BETA_T_CELL_DIFFERENTIATION	-2.0049336	0	0.001875508
GOBP_REGULATION_OF_CELL_KILLING	-2.0046449	0	0.001843925
GOBP_ADAPTIVE_IMMUNE_RESPONSE_BASED_ON_SOMATIC_RECOMBINATION_OF_IMMUNE_RECEPTORS_BUILT_FROM_IMMUNOGLOBULIN_SUPERFAMILY_DOMAINS	-2.0025353	0	0.001912281
GOBP_LYMPHOCYTE_HOMEOSTASIS	-1.9952343	0	0.002261707
GOBP_NATURAL_KILLER_CELL_MEDIATED_IMMUNITY	-1.9917516	0	0.00245388
GOBP_B_CELL_RECEPTOR_SIGNALING_PATHWAY	-1.9842315	0	0.00277038

GOBP_REGULATION_OF_CD4_POSITIVE_ALPHA_BETA_T_CELL_DIFFERENTIATION	-1.9840077	0	0.002697475
GOBP_LYMPHOCYTE_MEDIATED_IMMUNITY	-1.9826673	0	0.002692234
GOBP_LEUKOCYTE_PROLIFERATION	-1.9797264	0	0.002812522
GOBP_LEUKOCYTE_CELL_CELL_ADHESION	-1.9779865	0	0.002865658
GOBP_POSITIVE_REGULATION_OF_LEUKOCYTE_CELL_CELL_ADHESION	-1.9756947	0	0.002877024
GOBP_RESPONSE_TO_CHEMOKINE	-1.9754682	0	0.002829668
GOBP_REGULATION_OF_LEUKOCYTE_MEDIATED_CYTOTOXICITY	-1.9740149	0	0.002822431
GOBP_DENDRITIC_CELL_CHEMOTAXIS	-1.9727162	0	0.002833577
GOBP_T_CELL_SELECTION	-1.9726323	0	0.002771977
GOBP_IMMUNE_RESPONSE_REGULATING_CELL_SURFACE_RECEPTOR_SIGNALING_PATHWAY	-1.9678214	0	0.002908047
GOBP_LEUKOCYTE_MEDIATED_CYTOTOXICITY	-1.9643604	0	0.003125559
GOBP_POSITIVE_REGULATION_OF_NATURAL_KILLER_CELL_MEDIATED_CYTOTOXICITY	-1.9569992	0	0.003417886
GOBP_POSITIVE_REGULATION_OF_NATURAL_KILLER_CELL_MEDIATED_IMMUNITY	-1.951175	0	0.003699728
GOBP_T_CELL_DIFFERENTIATION	-1.9493088	0	0.003740826
GOBP_LYMPHOCYTE_CHEMOTAXIS	-1.9372544	0	0.004534826
GOBP_REGULATION_OF_NATURAL_KILLER_CELL_MEDIATED_IMMUNITY	-1.9306684	0	0.004983764
GOBP_FACE_DEVELOPMENT	-1.928116	0	0.005247035
GOBP_THYMIC_T_CELL_SELECTION	-1.9268554	0	0.005288136
GOBP_REGULATION_OF_ALPHA_BETA_T_CELL_DIFFERENTIATION	-1.9219942	0	0.005565974
GOBP_SENSORY_PERCEPTION_OF_SMELL	-1.9069043	0	0.006974414

GOBP_B_CELL_PROLIFERATION	-1.902837	0	0.007199158
GOBP_LEUKOCYTE_MEDIATED_IMMUNITY	-1.9000458	0	0.00747416
GOBP_T_CELL_ACTIVATION_INVOLVED_IN_IMMUNE_RESPONSE	-1.8987739	0	0.007502351
GOBP_T_CELL_DIFFERENTIATION_IN_THYMUS	-1.8987626	0	0.007379361
GOBP_T_CELL_HOMEOSTASIS	-1.8981528	0	0.007367177
GOBP_POSITIVE_REGULATION_OF_ALPHA_BETA_T_CELL_DIFFERENTIATION	-1.8960476	0.001324503	0.00742272
GOBP_LEUKOCYTE_HOMEOSTASIS	-1.8922163	0	0.007697986
GOBP_REGULATION_OF_LYMPHOCYTE_MEDIATED_IMMUNITY	-1.8735857	0	0.010187659
GOBP_T_CELL_MEDIATED_CYTOTOXICITY	-1.8730083	0	0.010134542
GOBP_FACE_MORPHOGENESIS	-1.8719716	0	0.01010685
GOBP_DENDRITIC_CELL_MIGRATION	-1.8718673	0	0.00995822
GOBP_T_CELL_MEDIATED_IMMUNITY	-1.8712368	0	0.009959191
GOBP_REGULATION_OF_ADAPTIVE_IMMUNE_RESPONSE	-1.8712295	0	0.009816918
GOBP_POSITIVE_REGULATION_OF_LYMPHOCYTE_ACTIVATION	-1.8702766	0	0.00981968
GOBP_SEQUESTERING_OF_CALCIUM_ION	-1.8683249	0	0.009983544
GOBP_NATURAL_KILLER_CELL_ACTIVATION	-1.8647677	0	0.010440228
GOBP_REGULATION_OF_T_CELL_DIFFERENTIATION	-1.8629161	0	0.010591991
GOBP_NEGATIVE_REGULATION_OF_ADAPTIVE_IMMUNE_RESPONSE	-1.8620546	0.001364257	0.01057329
GOBP_IMMUNE_EFFECTOR_PROCESS	-1.8598922	0	0.010895645
GOBP_POSITIVE_REGULATION_OF_CELL_ACTIVATION	-1.8596249	0	0.010786925
GOBP_REGULATION_OF_T_HELPER_17_TYPE_IMMUNE_RESPONSE	-1.8596165	0.001468429	0.01064863

GOBP_T_HELPER_17_CELL_DIFFERENTIATION	-1.859411	0	0.010535123
GOBP_REGULATION_OF_MYOBLAST_FUSION	-1.8572234	0.001515152	0.010756936
GOBP_MONONUCLEAR_CELL_DIFFERENTIATION	-1.8543103	0	0.010964353
GOBP_REGULATION_OF_LYMPHOCYTE_DIFFERENTIATION	-1.8539513	0	0.010891379
GOBP_IMMUNE_RESPONSE_REGULATING_SIGNALING_PATHWAY	-1.8527658	0	0.010941547
GOBP_MONOCYTE_CHEMOTAXIS	-1.849014	0.001369863	0.011466977
GOBP_REGULATION_OF_IMMUNE_EFFECTOR_PROCESS	-1.8444971	0	0.012166316
GOBP_CALCIUM_ION_TRANSMEMBRANE_IMPORT_INTO_CYTOSOL	-1.8377669	0	0.013313242
GOBP_NEGATIVE_REGULATION_OF_LYMPHOCYTE_ACTIVATION	-1.8334856	0	0.013937581
GOBP_REGULATION_OF_LEUKOCYTE_MEDIATED_IMMUNITY	-1.8320441	0	0.014053571
GOBP_INTERLEUKIN_2_PRODUCTION	-1.8318253	0	0.013932845
GOBP_POSITIVE_REGULATION_OF_CELL_CELL_ADHESION	-1.8305422	0	0.014010309
GOBP_TOLERANCE_INDUCTION	-1.8277252	0	0.014470044
GOBP_POSITIVE_REGULATION_OF_ANTIGEN_RECEPTOR_MEDIATED_SIGNALING_PATHWAY	-1.8267945	0	0.014548761
GOBP_NEGATIVE_REGULATION_OF_TISSUE_REMODELING	-1.8242415	0.001545595	0.014867924
GOBP_ESTABLISHMENT_OF_LYMPHOCYTE_POLARITY	-1.8221219	0.001512859	0.015241351
GOBP_INTERLEUKIN_12_PRODUCTION	-1.820969	0	0.015343821
GOBP_REGULATION_OF_T_CELL_MEDIATED_CYTOTOXICITY	-1.8194685	0	0.015505789
GOBP_REGULATION_OF_ANTIGEN_RECEPTOR_MEDIATED_SIGNALING_PATHWAY	-1.8175797	0.002663116	0.015715962
GOBP_B_CELL_ACTIVATION	-1.8121177	0	0.016824411

GOBP_T_CELL_DIFFERENTIATION_INVOLVED_IN_IMMUNE_RESPONSE	-1.8068947	0	0.01801926
GOBP_HEAD_MORPHOGENESIS	-1.8042399	0	0.018464297
GOBP_REGULATION_OF_B_CELL_PROLIFERATION	-1.8035024	0	0.018454585
GOBP_REGULATION_OF_INFLAMMATORY_RESPONSE	-1.8022201	0	0.018634304
GOBP_GAMMA_DELTA_T_CELL_ACTIVATION	-1.7954881	0.00148368	0.020315789
GOBP_NEUTROPHIL_CHEMOTAXIS	-1.7949858	0	0.020200651
GOBP_T_HELPER_1_TYPE_IMMUNE_RESPONSE	-1.7909344	0.004010695	0.021200635
GOBP_NEGATIVE_REGULATION_OF_CD4_POSITIVE_ALPHA_BETA_T_CELL_DIFFERENTIATION	-1.7877814	0.004267425	0.02192233
GOBP_COLLAGEN_METABOLIC_PROCESS	-1.7845693	0	0.02277858
GOBP_REGULATION_OF_T_CELL_RECEPTOR_SIGNALING_PATHWAY	-1.7779814	0.001362398	0.024576647
GOBP_LYMPHOCYTE_ACTIVATION_INVOLVED_IN_IMMUNE_RESPONSE	-1.777249	0	0.024581134
GOBP_NEGATIVE_REGULATION_OF_INTERLEUKIN_6_PRODUCTION	-1.7748438	0	0.025175488
GOBP_REGULATION_OF_T_HELPER_1_TYPE_IMMUNE_RESPONSE	-1.7747788	0.002853067	0.024971293
GOBP_POSITIVE_REGULATION_OF_INTERLEUKIN_12_PRODUCTION	-1.7731676	0.001381216	0.025254006
GOBP_NEUTROPHIL_MIGRATION	-1.7723147	0	0.025310358
GOBP_CYTOLYSIS	-1.7710947	0.004451039	0.025490167
GOBP_CELL_CELL_RECOGNITION	-1.7709988	0	0.025283016
GOBP_POSITIVE_REGULATION_OF_IMMUNE_EFFECTOR_PROCESS	-1.7708824	0	0.025108417
GOBP_POSITIVE_REGULATION_OF_B_CELL_PROLIFERATION	-1.7639664	0.002766252	0.02728256

GOBP_REGULATION_OF_T_CELL_MEDIATED_IMMUNITY	-1.7636145	0	0.027122106
GOBP_HUMORAL_IMMUNE_RESPONSE	-1.7631791	0	0.027048534
GOBP_PHOSPHATIDIC_ACID_METABOLIC_PROCESS	-1.7603822	0.004166667	0.027698215
GOBP_DEFENSE_RESPONSE_TO_GRAM_POSITIVE_BACTERIUM	-1.76001	0	0.027572673
GOBP_GRANULOCYTE_MIGRATION	-1.7586604	0	0.027778316
GOBP_LEUKOCYTE_CHEMOTAXIS	-1.7578917	0	0.027858421
GOBP_GRANULOCYTE_CHEMOTAXIS	-1.757636	0	0.027761431
GOBP_ACTIVATION_OF_IMMUNE_RESPONSE	-1.7546864	0	0.028560132
GOBP_NEGATIVE_REGULATION_OF_LEUKOCYTE_CELL_CELL_ADHESION	-1.7499943	0	0.030073777
GOBP_LYMPHOCYTE_MIGRATION	-1.7492678	0.001277139	0.030093307
GOBP_POSITIVE_REGULATION_OF_LYMPHOCYTE_MEDIATED_IMMUNITY	-1.7461681	0	0.031096248
GOBP_REGULATION_OF_LEUKOCYTE_APOPTOTIC_PROCESS	-1.7458882	0.00127551	0.030984454
GOBP_REGULATION_OF_T_CELL_DIFFERENTIATION_IN_THYMUS	-1.745748	0.002906977	0.030829571
GOBP_ANTIGEN_PROCESSING_AND_PRESENTATION_OF_ENDOGENOUS_PEPTIDE_ANTIGEN	-1.7456114	0.002932551	0.030670775
GOBP_NEUROMUSCULAR_SYNAPTIC_TRANSMISSION	-1.7441303	0.004398827	0.031032622
GOBP_RESPONSE_TO_PROTOZOAN	-1.7433885	0.004411765	0.031037545
GOBP_T_HELPER_1_CELL_DIFFERENTIATION	-1.7407005	0	0.031689275
GOBP_MAST_CELL_MEDIATED_IMMUNITY	-1.7359637	0.001362398	0.033291373
GOBP_NEGATIVE_REGULATION_OF_CYTOKINE_PRODUCTION_INVOLVED_IN_IMMUNE_RESPONSE	-1.7331964	0.002936858	0.03442749

GOBP_MYELOID_LEUKOCYTE_MIGRATION	-1.7308581	0	0.035169102
GOBP_CD4_POSITIVE_ALPHA_BETA_T_CELL_PROLIFERATION	-1.7302765	0.004291846	0.035168074
GOBP_NEGATIVE_REGULATION_OF_CELL_ACTIVATION	-1.7280178	0	0.0360192
GOBP_CELL_CHEMOTAXIS	-1.7270205	0	0.036262974
GOBP_B_CELL_DIFFERENTIATION	-1.7255101	0	0.036550023
GOBP_REGULATION_OF_FIBROBLAST_APOPTOTIC_PROCESS	-1.7254803	0.001485884	0.03629854
GOBP_REGULATION_OF_NEUROINFLAMMATORY_RESPONSE	-1.7201462	0.004178273	0.03849561
GOBP_POSITIVE_REGULATION_OF_TYPE_II_INTERFERON_PRODUCTION	-1.7187202	0.00131406	0.038865436
GOBP_REGULATION_OF_T_HELPER_CELL_DIFFERENTIATION	-1.7182381	0	0.038827304
GOBP_TYPE_II_INTERFERON_PRODUCTION	-1.718166	0	0.038589805
GOBP_FIBROBLAST_APOPTOTIC_PROCESS	-1.7154346	0.004195804	0.039785143
GOBP_POSITIVE_REGULATION_OF_CELL_ADHESION	-1.715288	0	0.03958418
GOBP_RESPONSE_TO_NITRIC_OXIDE	-1.7139584	0.005943536	0.03983999
GOBP_LEUKOCYTE_DIFFERENTIATION	-1.7134672	0	0.03983041
GOBP_PEPTIDE_CROSS_LINKING	-1.7132463	0.005899705	0.039732777
GOBP_POSITIVE_REGULATION_OF_T_CELL_RECEPTOR_SIGNALING_PATHWAY	-1.7130177	0	0.039569966
GOBP_NEGATIVE_REGULATION_OF_ALPHA_BETA_T_CELL_DIFFERENTIATION	-1.7125834	0.00729927	0.03949127
GOBP_NEGATIVE_REGULATION_OF_LEUKOCYTE_PROLIFERATION	-1.7118415	0.002583979	0.039521724
GOBP_NEGATIVE_REGULATION_OF_T_CELL_MEDIATED_IMMUNITY	-1.7117853	0.007163324	0.03928293
GOBP_INTERLEUKIN_17_PRODUCTION	-1.7095958	0.007042253	0.040088814
GOBP_POSITIVE_REGULATION_OF_LYMPHOCYTE_CHEMOTAXIS	-1.7068042	0.007451565	0.04114579

GOBP_CELL_ACTIVATION_INVOLVED_IN_IMMUNE_RESPONSE	-1.7062833	0	0.041176144
GOBP_INTEGRIN_ACTIVATION	-1.7036756	0.004310345	0.04220802
GOBP_NEGATIVE_REGULATION_OF_CD4_POSITIVE_ALPHA_BETA_T_CELL_ACTIVATION	-1.7005819	0.005788712	0.043445095
GOBP_LEUKOCYTE_APOPTOTIC_PROCESS	-1.6985251	0	0.044362742
GOBP_REGULATION_OF_CD8_POSITIVE_ALPHA_BETA_T_CELL_ACTIVATION	-1.6940473	0.002919708	0.046344567
GOBP_HETEROPHILIC_CELL_CELL_ADHESION_VIA_PLASMA_MEMBRANE_CELL_ADHESION_MOLECULES	-1.6919544	0.009708738	0.047253016
GOBP_NEGATIVE_REGULATION_OF_LEUKOCYTE_CHEMOTAXIS	-1.6919441	0.006079027	0.04697
GOBP_REGULATION_OF_LEUKOCYTE_DEGRANULATION	-1.6917671	0.00270636	0.046786524
GOBP_POSITIVE_REGULATION_OF_LEUKOCYTE_MEDIATED_IMMUNITY	-1.6900338	0.001213592	0.047444984
GOBP_ARACHIDONIC_ACID_SECRETION	-1.6851263	0.00862069	0.049874187

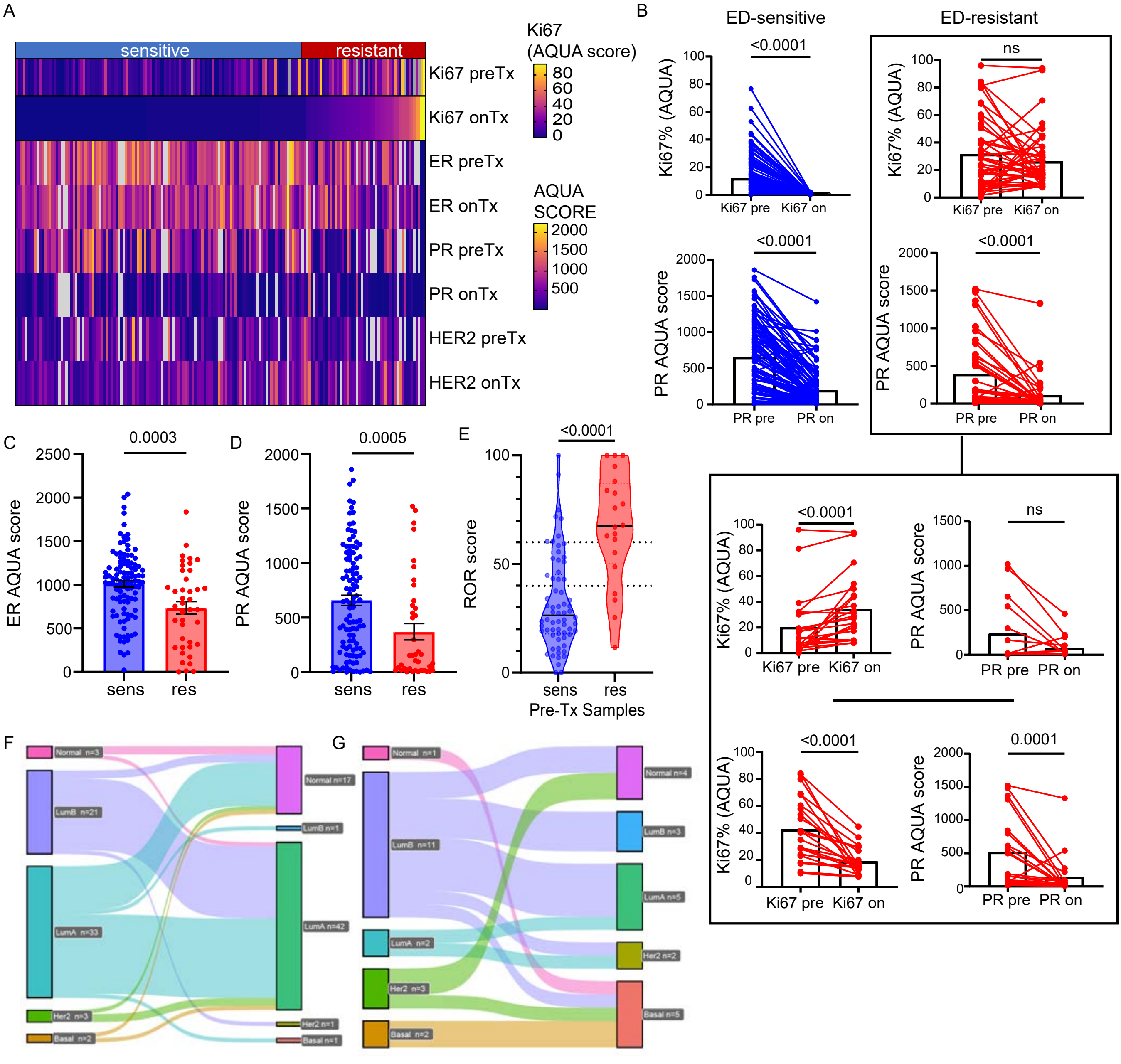
A
sensitive
resistant
Ki67 preTx
Ki67 onTx
ER preTx
ER onTx
PR preTx
PR onTx
HER2 preTx
HER2 onTx
Ki67
(AQUA score)
80
60
40
20
0
AQUA
SCORE
2000
1500
1000
500
B
ED-sensitive
ED-resistant
<0.0001
Ki67% (AQUA)
Ki67 pre
Ki67 on
ns
PR AQUA score
PR pre
PR on
C
0.0003
ER AQUA score
sens
res
D
0.0005
PR AQUA score
E
<0.0001
ROR score
Pre-Tx Samples
0.0001
F
Normal n=3
LumB n=21
LumA n=33
Her2 n=3
Basal n=2
Normal n=17
LumB n=1
LumA n=42
Her2 n=1
Basal n=1
G
Normal n=1
LumB n=11
LumA n=2
Her2 n=3
Basal n=2
Normal n=4
LumB n=3
LumA n=5
Her2 n=2
Basal n=5

Supplemental Figure 1.

(A) Heatmap of Ki67, ER, PR, and HER2 scores using AQUA. Samples are shown in order of Ki67 scores in the onTx tumors from low on the left to high on the right. (B) Dot plot of Ki67 scores and PR expression (blue, ED-sensitive; red, ED-resistant). In the highlighted square, Ki67 and PR from the ED-resistant group have been divided based on Ki67 modification: in the upper part, tumors whose Ki67 did not change or increased, on the bottom the tumors whose Ki67 decreased but without crossing the 7.4% cutoff. Comparisons were made using paired t-test. (C and D) Comparison of baseline levels of ER (C) and PR (D), in ED-sensitive vs. -resistant tumors. Unpaired t-test was used for comparison. (E) ROR scores were extracted using RNA-Seq derived gene expression data as described in Methods. Unpaired t-test was used for comparison. (F and G) Sankey plots of ED-sensitive (F) and ED-resistant (G) tumors, showing the modification of the PAM50 distribution from preTx, on the left, to onTx, on the right.

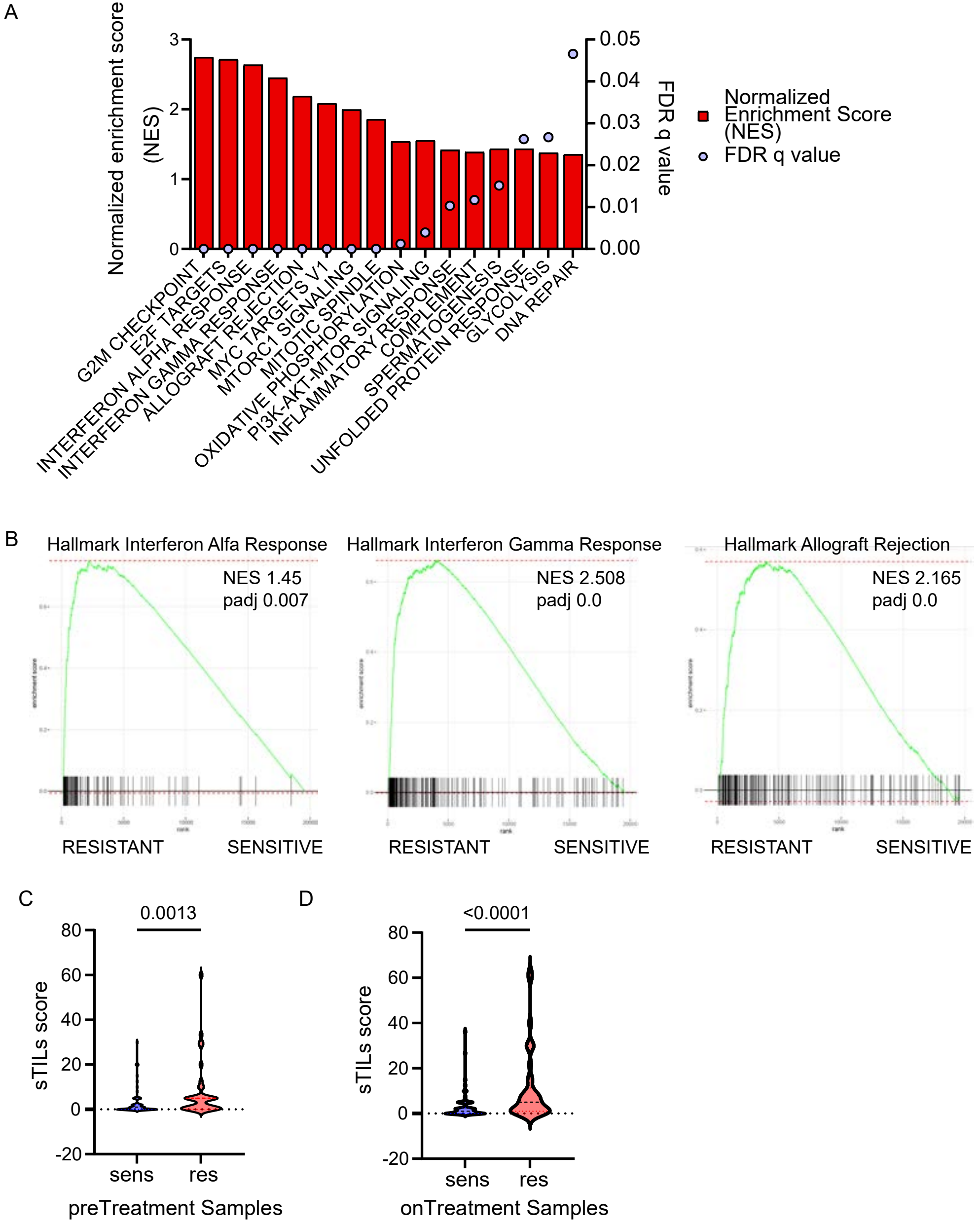
A
Normalized enrichment score (NES)
FDR q value
3
2
1
0
0.05
0.04
0.03
0.02
0.01
0.00
Normalized Enrichment Score (NES)
FDR q value
G2M CHECKPOINT
E2F TARGETS
INTERFERON ALPHA RESPONSE
INTERFERON GAMMA RESPONSE
ALLOGRAFT REJECTION
MYC TARGETS V1
MTORC1 SIGNALING
MITOTIC SPINDLE
OXIDATIVE PHOSPHORYLATION
PI3K-AKT-MTOR SIGNALING
INFLAMMATORY RESPONSE
COMPLEMENT
SPERMATOGENESIS
UNFOLDED PROTEIN RESPONSE
GLYCOLYSIS
DNA REPAIR
B
Hallmark Interferon Alfa Response
NES 1.45
padj 0.007
RESISTANT
SENSITIVE
Hallmark Interferon Gamma Response
NES 2.508
padj 0.0
RESISTANT
SENSITIVE
Hallmark Allograft Rejection
NES 2.165
padj 0.0
RESISTANT
SENSITIVE
C
0.0013
sTILs score
80
60
40
20
0
-20
sens
res
preTreatment Samples
D
<0.0001
sTILs score
80
60
40
20
0
-20
sens
res
onTreatment Samples

Supplemental Figure 2.

(A) Gene set enrichment analysis (GSEA). Bar plot shows the enrichment scores and q-values of the gene signatures upregulated in ED-resistant vs. ED-sensitive tumors at the time of surgery. (B) Selected GSEA plots from (A). (C and D) Violin plots of sTIL scores evaluated on H&E slides in preTx (C) and onTx (D) tumors as described in Methods. Comparisons were made using unpaired t-test.

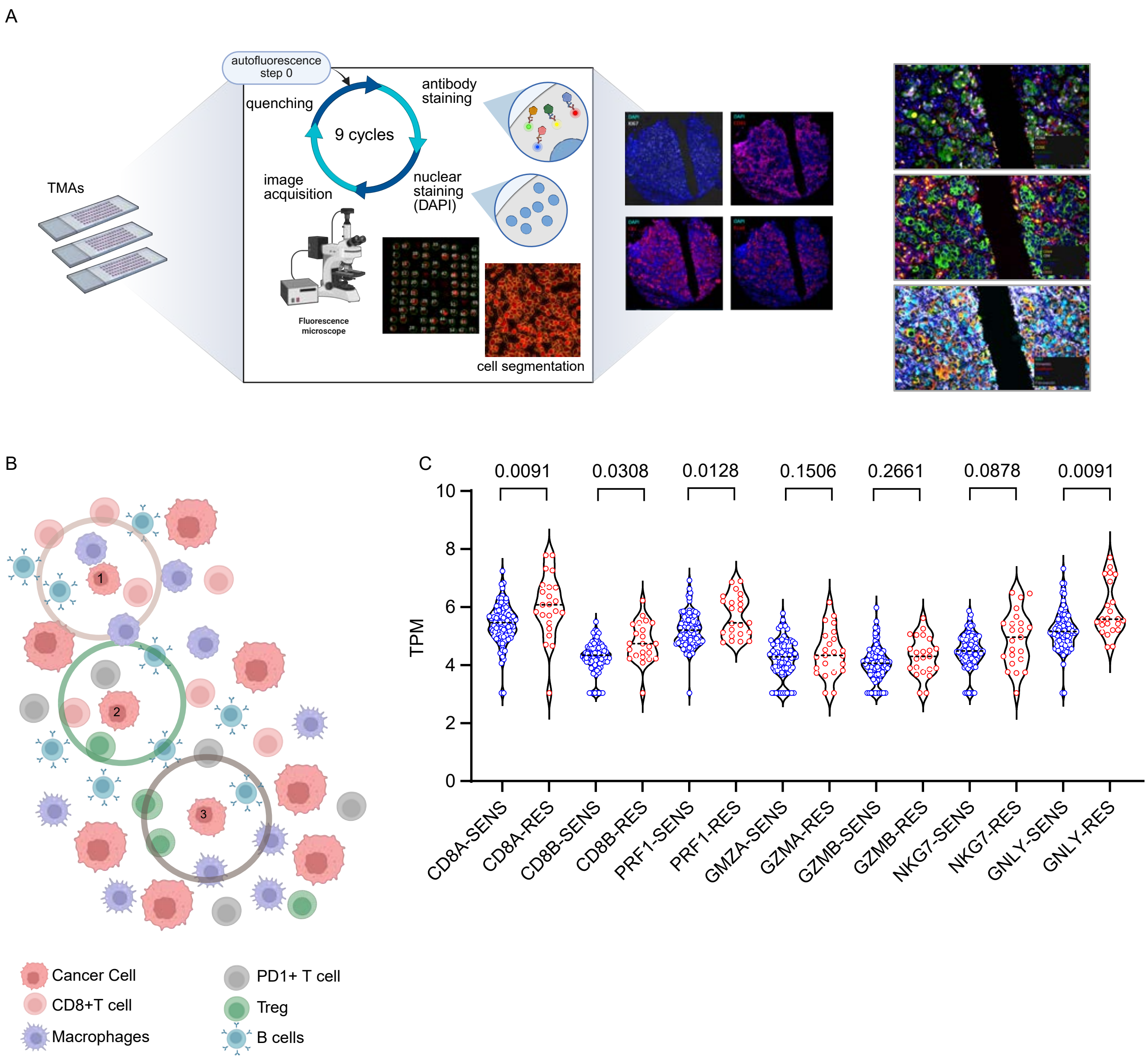
A
autofluorescence step 0
quenching
9 cycles
antibody staining
nuclear staining (DAPI)
image acquisition
TMAs
Fluorescence microscope
cell segmentation
B
1
2
3
Cancer Cell
CD8+T cell
Macrophages
PD1+ T cell
Treg
B cells
C
0.0091
0.0308
0.0128
0.1506
0.2661
0.0878
0.0091
TPM
10
8
6
4
2
0
CD8A-SENS
CD8A-RES
CD8B-SENS
CD8B-RES
PRF1-SENS
PRF1-RES
GMZA-SENS
GZMA-RES
GZMB-SENS
GZMB-RES
NKG7-SENS
NKG7-RES
GNLY-SENS
GNLY-RES

Supplemental Figure 3.

(A) Schema of the cyclic immunofluorescence (CycIF) method. TMAs were prepared from 174 FFPE blocks of onTx samples (n=118 patients). A total of 38 immunofluorescent antibodies were used (see Methods). After acquiring the images and performing cell segmentation on each tissue, the cells positive for E-cadherin or Pan-cytokeratin were labeled as cancer cells. On the right, examples of images acquired using CycIF. (B) Schema of the approach used to characterize the expression of CycIF markers in the “pressure area" (see Methods). (C) Expression of genes associated with CD8+ T cells cytolytic activity, in ED-resistant vs. ED-sensitive tumors evaluated in the on-treatment samples. Unpaired t-test was used for comparison.

New Cohort	
RESISTANT SAMPLES	SENSITIVE SAMPLES
4 pre-treatment<br>6 on-treatment<br>[4 samples are matched]	4 pre-treatment<br>6 on-treatment<br>[4 samples are matched]

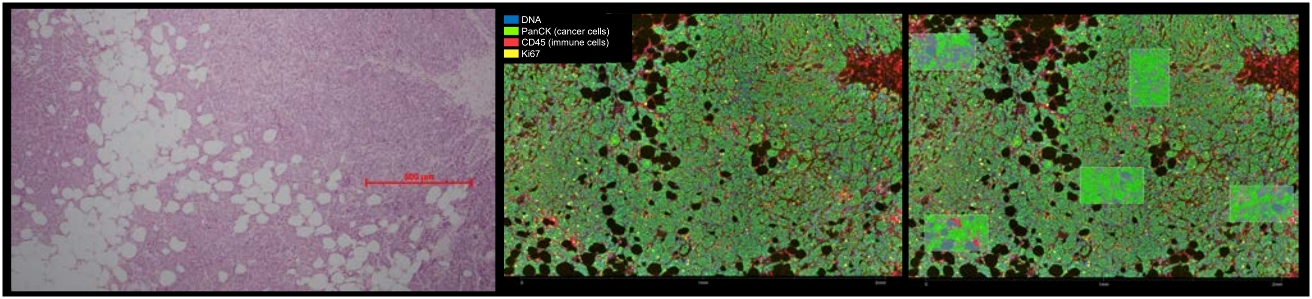

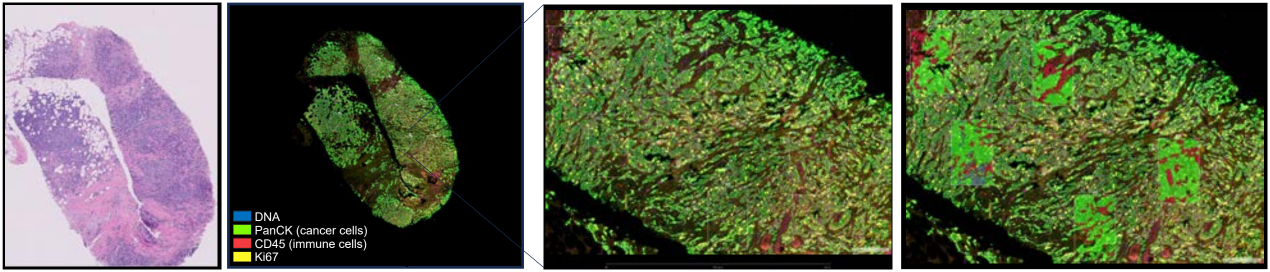

Supplemental Figure 4.

Example of tumor sections profiled with GeoMx®. The legend for each marker's color is on the side. Top panel: On treatment tumor (surgically resected). Bottom panel: Tumor before treatment. Top panel from left to right: H&E image; IF image after staining with DNA, PanCK, CD45, and Ki67 markers; selection of the regions of interest (ROIs). Bottom panel from left to right: H&E image; IF image after staining DNA, PanCK, CD45, and Ki67 markers; a detail of the tumor section; last panel on the right shows how the software identified ROIs with cancer (panCK+/CD45-negative) and immune cells (panCK-negative/CD45+) used for transcriptomic sequencing.

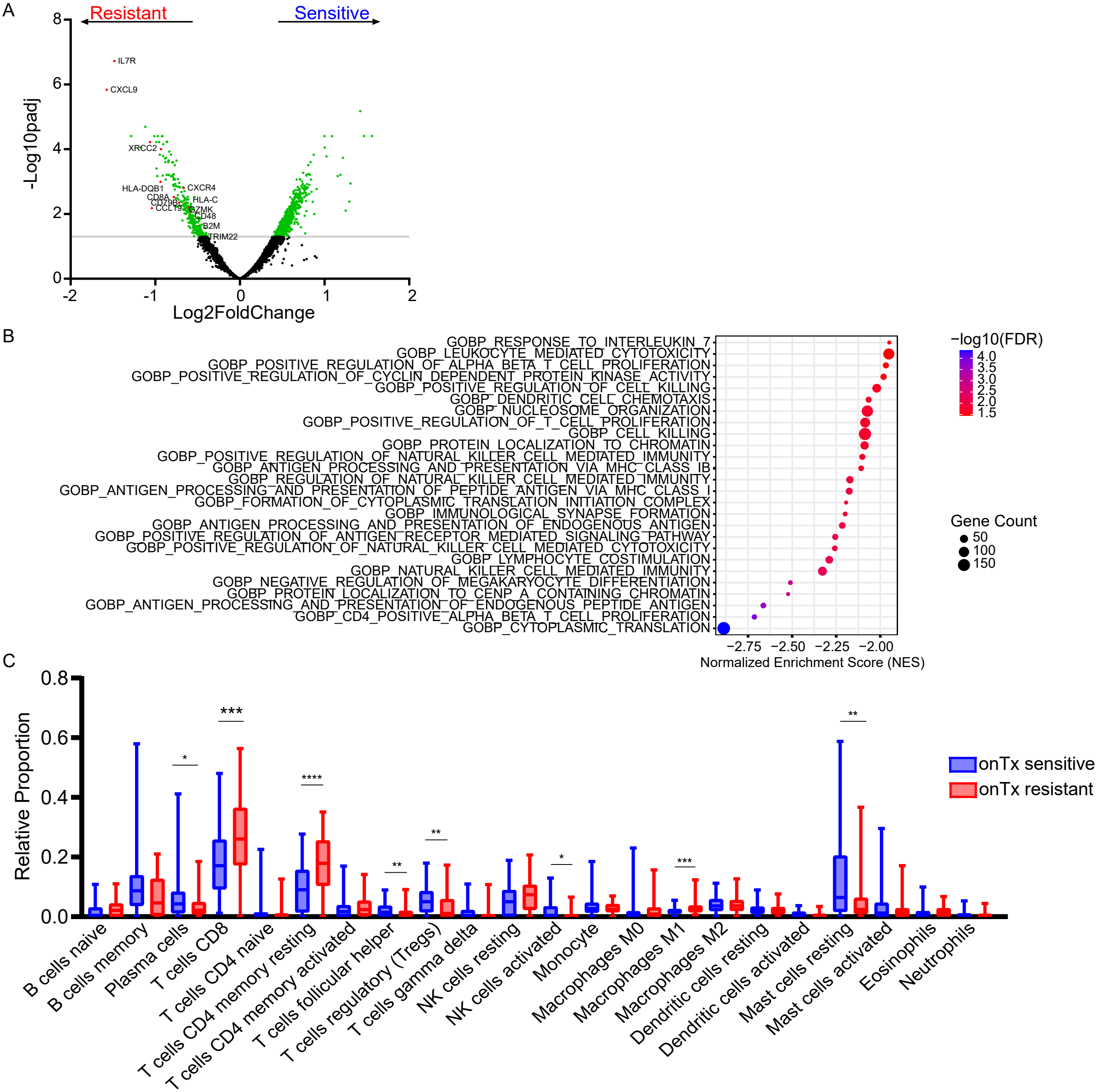
A
Resistant
Sensitive
-Log10padj
Log2FoldChange
IL7R
CXCL9
XRCC2
HLA-DQB1
CXCR4
CD8A
CD79B
HLA-C
CCL19
GZMK
CD48
B2M
TRIM22
B
GOBP_RESPONSE_TO_INTERLEUKIN_7
GOBP_LEUKOCYTE_MEDIATED_CYTOTOXICITY
GOBP_POSITIVE_REGULATION_OF_ALPHA_BETA_T_CELL_PROLIFERATION
GOBP_POSITIVE_REGULATION_OF_CYCLIN_DEPENDENT_PROTEIN_KINASE_ACTIVITY
GOBP_POSITIVE_REGULATION_OF_CELL_KILLING
GOBP_DENDRITIC_CELL_CHEMOTAXIS
GOBP_NUCLEOSOME_ORGANIZATION
GOBP_POSITIVE_REGULATION_OF_T_CELL_PROLIFERATION
GOBP_CELL_KILLING
GOBP_PROTEIN_LOCALIZATION_TO_CHROMATIN
GOBP_POSITIVE_REGULATION_OF_NATURAL_KILLER_CELL_MEDIATED_IMMUNITY
GOBP_ANTIGEN_PROCESSING_AND_PRESENTATION_VIA_MHC_CLASS_IB
GOBP_REGULATION_OF_NATURAL_KILLER_CELL_MEDIATED_IMMUNITY
GOBP_ANTIGEN_PROCESSING_AND_PRESENTATION_OF_PEPTIDE_ANTIGEN_VIA_MHC_CLASS_I
GOBP_FORMATION_OF_CYTOPLASMIC_TRANSLATION_INITIATION_COMPLEX
GOBP_IMMUNOLOGICAL_SYNAPSE_FORMATION
GOBP_ANTIGEN_PROCESSING_AND_PRESENTATION_OF_ENDOGENOUS_ANTIGEN
GOBP_POSITIVE_REGULATION_OF_ANTIGEN_RECEPTOR_MEDIATED_SIGNALING_PATHWAY
GOBP_POSITIVE_REGULATION_OF_NATURAL_KILLER_CELL_MEDIATED_CYTOTOXICITY
GOBP_LYMPHOCYTE_COSTIMULATION
GOBP_NATURAL_KILLER_CELL_MEDIATED_IMMUNITY
GOBP_NEGATIVE_REGULATION_OF_MEGAKARYOCYTE_DIFFERENTIATION
GOBP_PROTEIN_LOCALIZATION_TO_CENP_A_CONTAINING_CHROMATIN
GOBP_ANTIGEN_PROCESSING_AND_PRESENTATION_OF_ENDOGENOUS_PEPTIDE_ANTIGEN
GOBP_CD4_POSITIVE_ALPHA_BETA_T_CELL_PROLIFERATION
GOBP_CYTOPLASMIC_TRANSLATION
Normalized Enrichment Score (NES)
−log10(FDR)
Gene Count
C
Relative Proportion
onTx sensitive
onTx resistant
B cells naive
B cells memory
Plasma cells
T cells CD8
T cells CD4 naive
T cells CD4 memory resting
T cells CD4 memory activated
T cells follicular helper
T cells regulatory (Tregs)
T cells gamma delta
NK cells resting
NK cells activated
Monocyte
Macrophages M0
Macrophages M1
Macrophages M2
Dendritic cells resting
Dendritic cells activated
Mast cells resting
Mast cells activated
Eosinophils
Neutrophils

Supplemental Figure 5.

(A) Volcano plot showing differentially expressed genes in the CD45+ (immune) cell compartment. Genes on the left side of the plot are enriched in ED-resistant tumors. (B) Gene Ontology Biology Process analysis performed using the genes in (A). (C) Comparison of immune cell distribution in ED-sensitive vs. ED-resistant tumors, estimated using CIBERSORT in onTx samples. Wilcoxon rank-sum test was applied to compare cell type distributions; $p<0.05$ was considered statistically significant (*, $p<0.05$, **, $p<0.01$, ***, $p<0.001$, ****, $p<0.0001$).

A

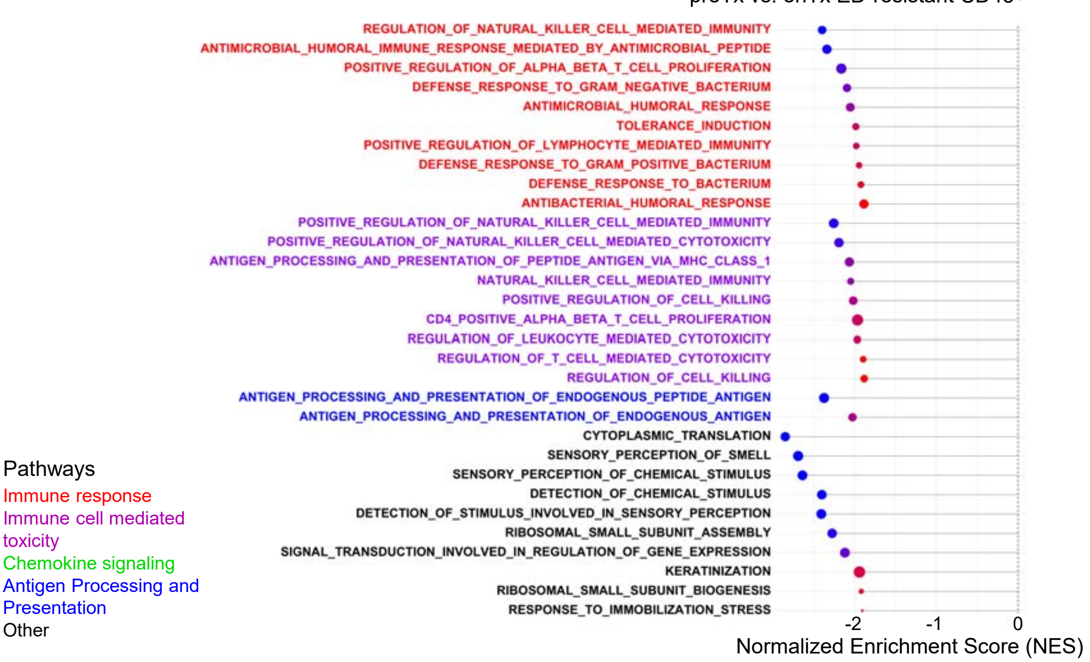

preTx vs. onTx ED-resistant CD45+
REGULATION_OF_NATURAL_KILLER_CELL_MEDIATED_IMMUNITY
ANTIMICROBIAL_HUMORAL_IMMUNE_RESPONSE_MEDIATED_BY_ANTIMICROBIAL_PEPTIDE
POSITIVE_REGULATION_OF_ALPHA_BETA_T_CELL_PROLIFERATION
DEFENSE_RESPONSE_TO_GRAM_NEGATIVE_BACTERIUM
ANTIMICROBIAL_HUMORAL_RESPONSE
TOLERANCE_INDUCTION
POSITIVE_REGULATION_OF_LYMPHOCYTE_MEDIATED_IMMUNITY
DEFENSE_RESPONSE_TO_GRAM_POSITIVE_BACTERIUM
DEFENSE_RESPONSE_TO_BACTERIUM
ANTIBACTERIAL_HUMORAL_RESPONSE
POSITIVE_REGULATION_OF_NATURAL_KILLER_CELL_MEDIATED_IMMUNITY
POSITIVE_REGULATION_OF_NATURAL_KILLER_CELL_MEDIATED_CYTOTOXICITY
ANTIGEN_PROCESSING_AND_PRESENTATION_OF_PEPTIDE_ANTIGEN_VIA_MHC_CLASS_1
NATURAL_KILLER_CELL_MEDIATED_IMMUNITY
POSITIVE_REGULATION_OF_CELL_KILLING
CD4_POSITIVE_ALPHA_BETA_T_CELL_PROLIFERATION
REGULATION_OF_LEUKOCYTE_MEDIATED_CYTOTOXICITY
REGULATION_OF_T_CELL_MEDIATED_CYTOTOXICITY
REGULATION_OF_CELL_KILLING
ANTIGEN_PROCESSING_AND_PRESENTATION_OF_ENDOGENOUS_PEPTIDE_ANTIGEN
ANTIGEN_PROCESSING_AND_PRESENTATION_OF_ENDOGENOUS_ANTIGEN
CYTOPLASMIC_TRANSLATION
SENSORY_PERCEPTION_OF_SMELL
SENSORY_PERCEPTION_OF_CHEMICAL_STIMULUS
DETECTION_OF_CHEMICAL_STIMULUS
DETECTION_OF_STIMULUS_INVOLVED_IN_SENSORY_PERCEPTION
RIBOSOMAL_SMALL_SUBUNIT_ASSEMBLY
SIGNAL_TRANSDUCTION_INVOLVED_IN_REGULATION_OF_GENE_EXPRESSION
KERATINIZATION
RIBOSOMAL_SMALL_SUBUNIT_BIOGENESIS
RESPONSE_TO_IMMOBILIZATION_STRESS
-2
-1
0
Normalized Enrichment Score (NES)
Pathways
Immune response
Immune cell mediated toxicity
Chemokine signaling
Antigen Processing and Presentation
Other

B

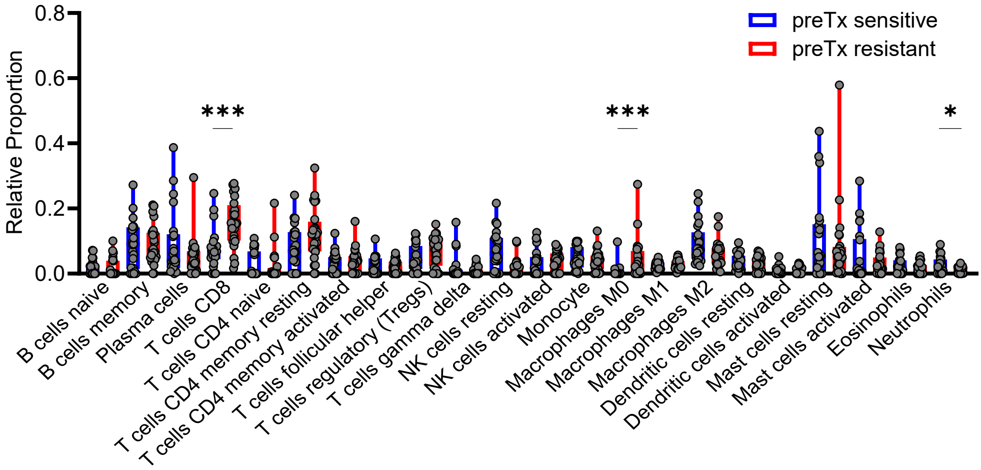

0.8
0.6
0.4
0.2
0.0
Relative Proportion
preTx sensitive
preTx resistant
***
***
*
B cells naive
B cells memory
Plasma cells
T cells CD8
T cells CD4 naive
T cells CD4 memory resting
T cells CD4 memory activated
T cells follicular helper
T cells regulatory (Tregs)
T cells gamma delta
NK cells resting
NK cells activated
Monocyte
Macrophages M0
Macrophages M1
Macrophages M2
Dendritic cells resting
Dendritic cells activated
Mast cells resting
Mast cells activated
Eosinophils
Neutrophils

Supplemental Figure 6.

(A) List of the top enriched pathways enriched in CD45+ cells, in onTx vs. preTx ED-resistant tumors. (B) Comparison of immune cell distribution in preTx ED-sensitive vs preTx ED-resistant tumors, estimated using CIBERSORT. Wilcoxon rank-sum test was applied to compare cell type distributions; $p<0.05$ was considered statistically significant (*, $p<0.05$, **, $p<0.01$, ***, $p<0.001$, ****, $p<0.0001$).

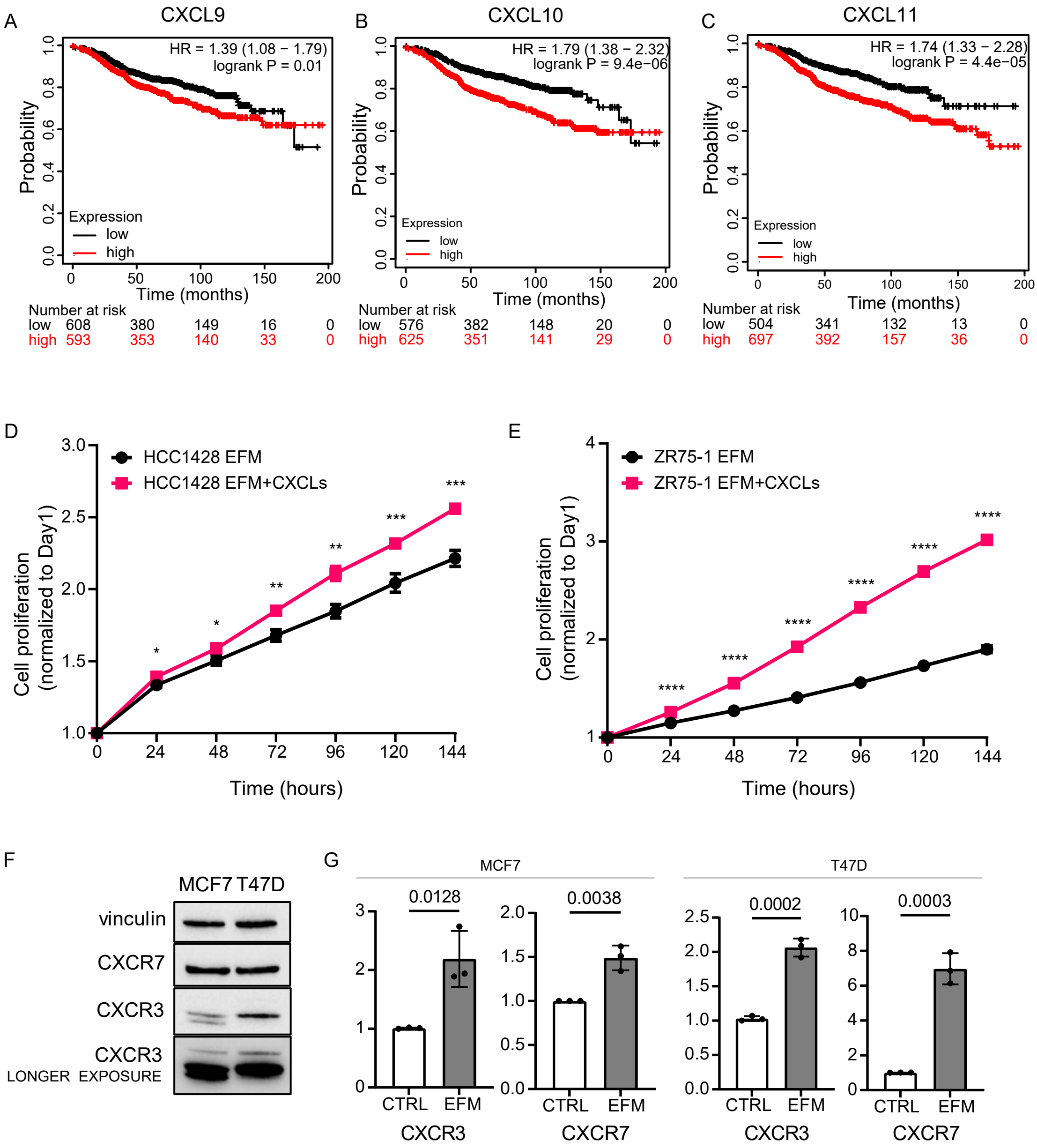

A
CXCL9
HR = 1.39 (1.08 − 1.79)
logrank P = 0.01
Probability
Expression
low
high
Time (months)
Number at risk
low 608 380 149 16 0
high 593 353 140 33 0
B
CXCL10
HR = 1.79 (1.38 − 2.32)
logrank P = 9.4e−06
Probability
Expression
low
high
Time (months)
Number at risk
low 576 382 148 20 0
high 625 351 141 29 0
C
CXCL11
HR = 1.74 (1.33 − 2.28)
logrank P = 4.4e−05
Probability
Expression
low
high
Time (months)
Number at risk
low 504 341 132 13 0
high 697 392 157 36 0
D
HCC1428 EFM
HCC1428 EFM+CXCLs
Cell proliferation (normalized to Day1)
Time (hours)
E
ZR75-1 EFM
ZR75-1 EFM+CXCLs
Cell proliferation (normalized to Day1)
Time (hours)
F
MCF7 T47D
vinculin
CXCR7
CXCR3
CXCR3
LONGER EXPOSURE
G
MCF7
T47D
0.0128
0.0038
0.0002
0.0003
CTRL EFM
CXCR3
CXCR7

Supplemental Figure 7.

(A-C) Kaplan-Meier curves comparing the RFS probability of patients with HR+/HER2-negative breast cancer treated with endocrine therapy (kmplot.com) with high vs. low expression of CXCL9 (A), CXCL10 (B), and CXCL11 (C). (D and E) HCC1428 (D) and ZR75-1 (E) were seeded in estrogen free media. CXCL9, CXCL10 and CXCL11 (10 nM each) were added the following day. Cell proliferation was assessed daily on days 1-7 with Incucyte. Unpaired t-test was used to compare cell proliferation between the cytokine arms and untreated controls. Mean ± SD of no. cells are shown (SDs not always visible when smaller than the size of the symbol). Statistical analysis was done by unpaired t-test (*, $p<0.05$, **, $p<0.01$, ***, $p<0.001$, ****, $p<0.0001$). (F) Immunoblot analysis of MCF7 and T47D lysates. The cells were seeded in full medium; the lysates were collected and probed with the indicated antibodies. (G) RT-PCR of CXCR3 and CXCR7 receptors in MCF7 and T47D. RNA was extracted from cells seeded in full medium or estrogen-free medium after 48hours. Unpaired t-test was used for comparison. $p<0.05$ was considered statistically significant. P-values are shown in the figure.

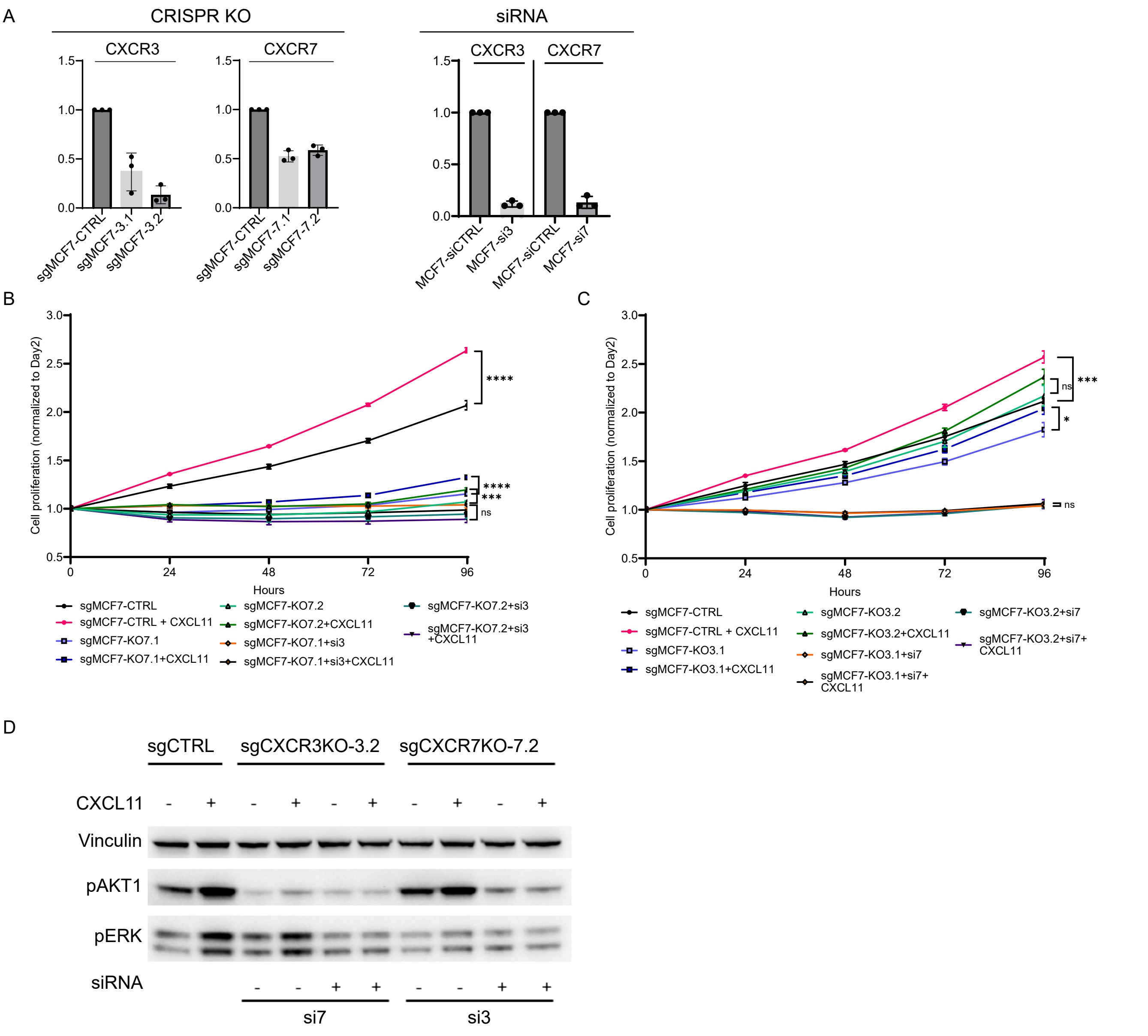
A
CRISPR KO
siRNA
CXCR3
CXCR7
sgMCF7-CTRL
sgMCF7-3.1
sgMCF7-3.2
sgMCF7-7.1
sgMCF7-7.2
MCF7-siCTRL
MCF7-si3
MCF7-si7
B
C
Cell proliferation (normalized to Day2)
Hours
sgMCF7-CTRL
sgMCF7-CTRL + CXCL11
sgMCF7-KO7.1
sgMCF7-KO7.1+CXCL11
sgMCF7-KO7.2
sgMCF7-KO7.2+CXCL11
sgMCF7-KO7.1+si3
sgMCF7-KO7.1+si3+CXCL11
sgMCF7-KO7.2+si3
sgMCF7-KO7.2+si3 +CXCL11
sgMCF7-KO3.1
sgMCF7-KO3.1+CXCL11
sgMCF7-KO3.2
sgMCF7-KO3.2+CXCL11
sgMCF7-KO3.1+si7
sgMCF7-KO3.1+si7+ CXCL11
sgMCF7-KO3.2+si7
sgMCF7-KO3.2+si7+ CXCL11
D
sgCTRL
sgCXCR3KO-3.2
sgCXCR7KO-7.2
CXCL11
Vinculin
pAKT1
pERK
siRNA
si7
si3

Supplemental Figure 8.

(A) qRT-PCR was used to assess the efficacy of CRISPR KO (on the left) and siRNA (right). (B) Growth curve of MCF7-sgCTRL vs. -sgKO3.1 vs -sgKO3.2, with or without CXCL11 10nM, with or without si7 (siRNA against CXCR7). Proliferation data were acquired using Incucyte, with images every 24h. (C) Growth curve of MCF7-sgCTRL vs. -sgKO7.1 vs -sgKO7.2, ± CXCL11 10nM ± si3 (siRNA against CXCR3). Proliferation data were acquired using Incucyte, with images every 24h. (D) Immunoblot analysis of MCF7-sgCTRL ± CXCL11, vs MCF7-sgKO3.2 ± CXCL11 ± si7, vs MCF7-sgKO7.2 ± CXCL11 ± si3. The cells were seeded overnight in serum starvation and the lysates were collected 15 minutes after adding CXCL11 10 nM, and then probed with the indicated antibodies.

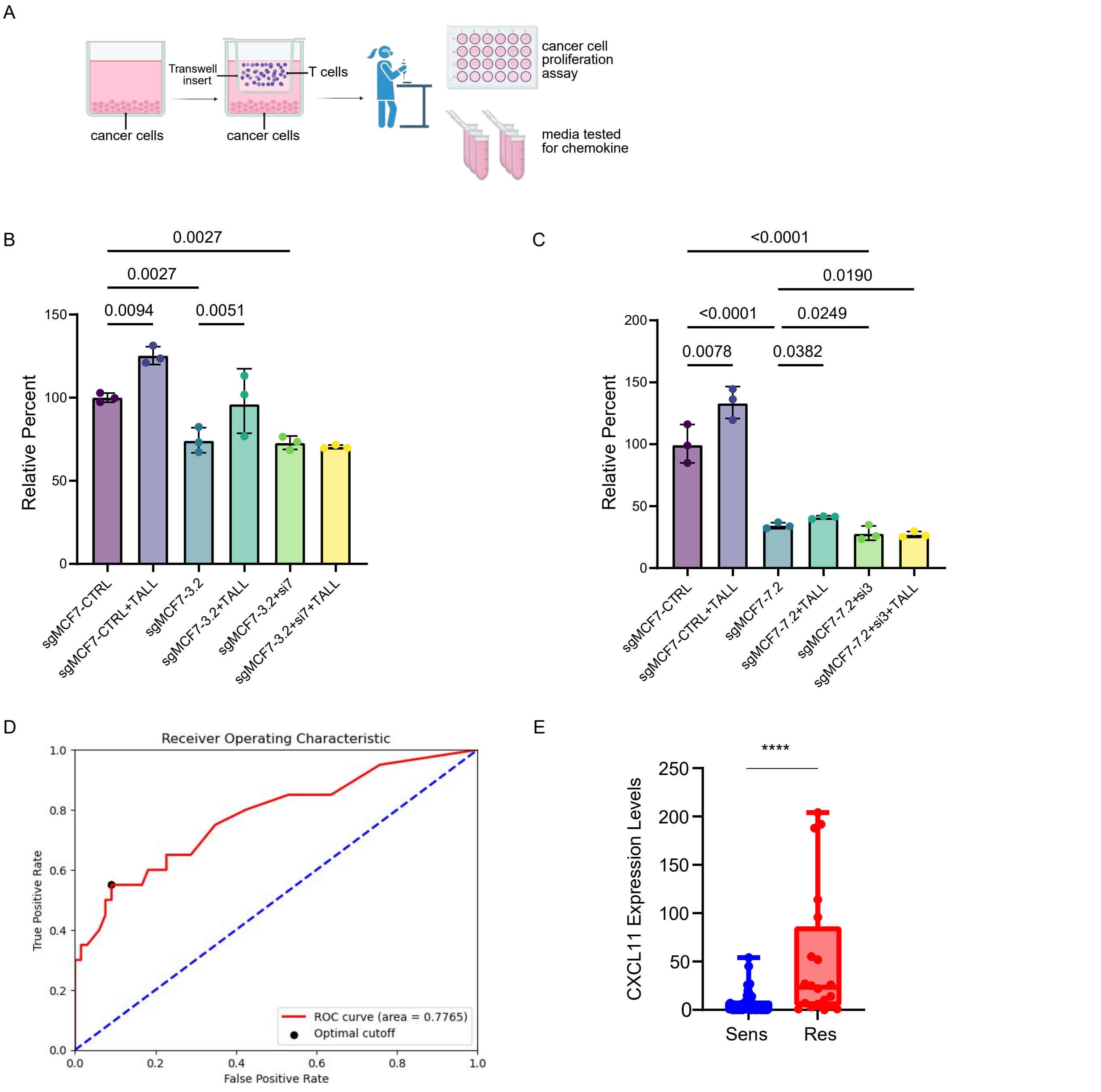
A
Transwell insert
T cells
cancer cells
cancer cells
cancer cell proliferation assay
media tested for chemokine
B
0.0027
0.0027
0.0094
0.0051
Relative Percent
150
100
50
0
sgMCF7-CTRL
sgMCF7-CTRL+TALL
sgMCF7-3.2
sgMCF7-3.2+TALL
sgMCF7-3.2+si7
sgMCF7-3.2+si7+TALL
C
<0.0001
0.0190
<0.0001
0.0249
0.0078
0.0382
Relative Percent
200
150
100
50
0
sgMCF7-CTRL
sgMCF7-CTRL+TALL
sgMCF7-7.2
sgMCF7-7.2+TALL
sgMCF7-7.2+si3
sgMCF7-7.2+si3+TALL
D
Receiver Operating Characteristic
True Positive Rate
False Positive Rate
0.0
0.2
0.4
0.6
0.8
1.0
ROC curve (area = 0.7765)
Optimal cutoff
E
****
CXCL11 Expression Levels
250
200
150
100
50
0
Sens
Res

Supplemental Figure 9.

(A) Schematic representation of co-culture experiments. On the first day, 3×104 MCF7 cells were seeded in triplicate in multi-wells in estrogen-free media. After 12h, transwells containing 10,000 or 20,000 T-ALL 104 cells + IL2 were added. MCF7 cells without co-cultured T ALL-104 cells were also treated with IL2. After 6 days, the conditioned media was collected and subjected to Cytokine ELISA. (B) sgMCF7-CTRL, sgMCF7-3.2, sgMCF7-3.2 ± si7 cells were seeded in EFM ± T-ALL 104 cells. The cancer cell number was evaluated 6 days after initial seeding. Experiments were performed at least three times in triplicate wells. Statistical differences were assessed using one-way ANOVA with FDR correction for multiple testing; q<0.05 was considered statistically significant. (C) sgMCF7-CTRL, sgMCF7-7.2, sgMCF7-7.2 ± si3 cells were seeded in EFM ± T-ALL 104 cells. The cancer cell number was evaluated 6 days after initial seeding. Experiments were performed at least three times in triplicate wells. Statistical differences were assessed using one-way ANOVA with FDR correction for multiple testing; q<0.05 was considered statistically significant. (D) ROC curve (receiver operating characteristic curve) shows the performance of CXCL11 RNA levels in predicting sensitivity to endocrine treatment in patients (n=86) in the clinical trial; AUC was 0.777. (E) Boxplot of CXCL11 expression levels by response. Statistical analysis was done by chi-square test (p=7.038×10-7).

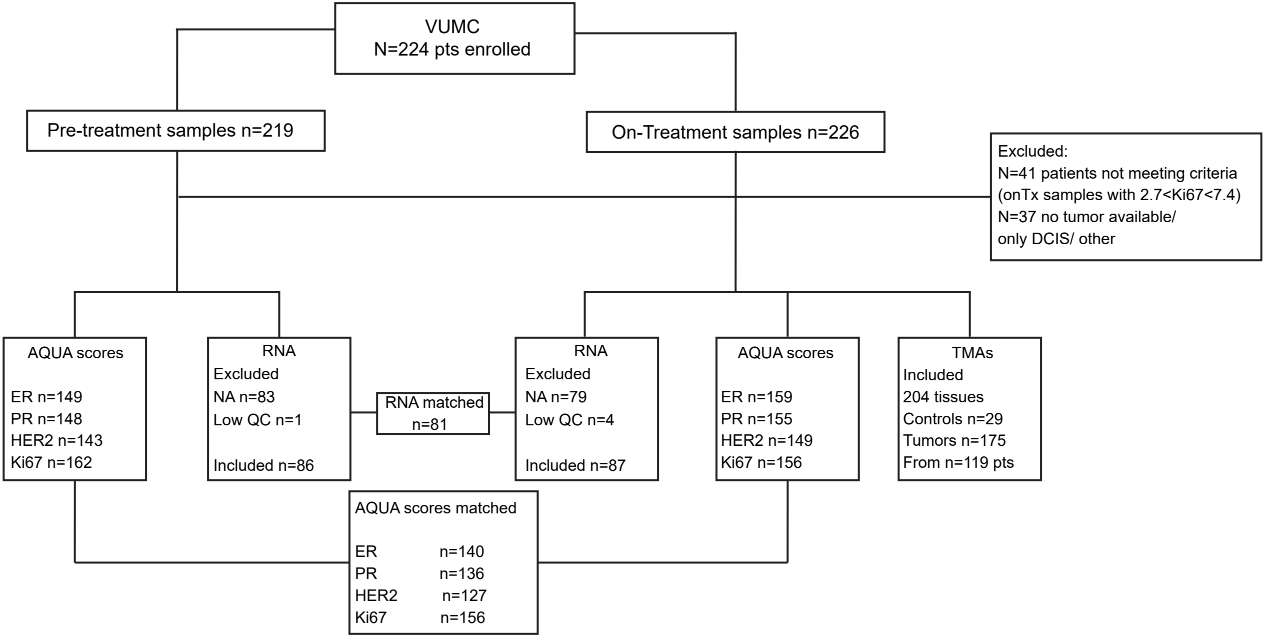

Supplemental Figure 10.
Consort diagram of the patients erolled in the VUMC cohort.

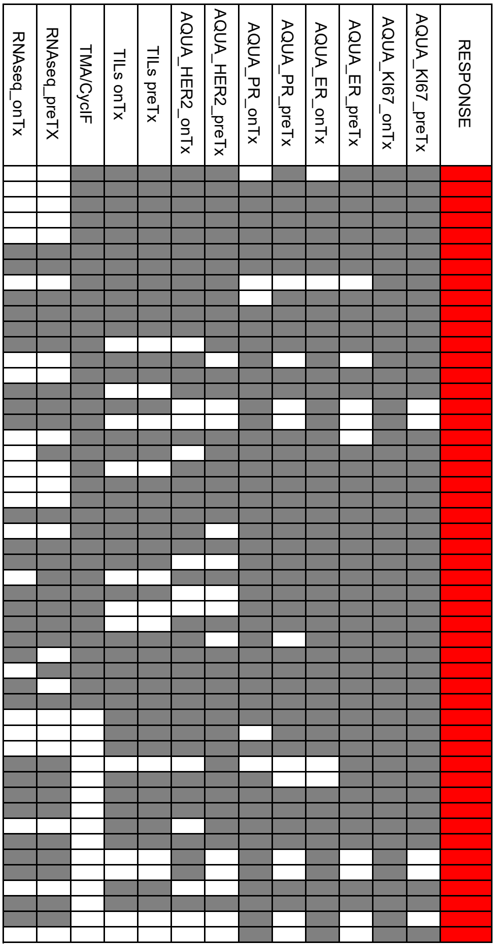

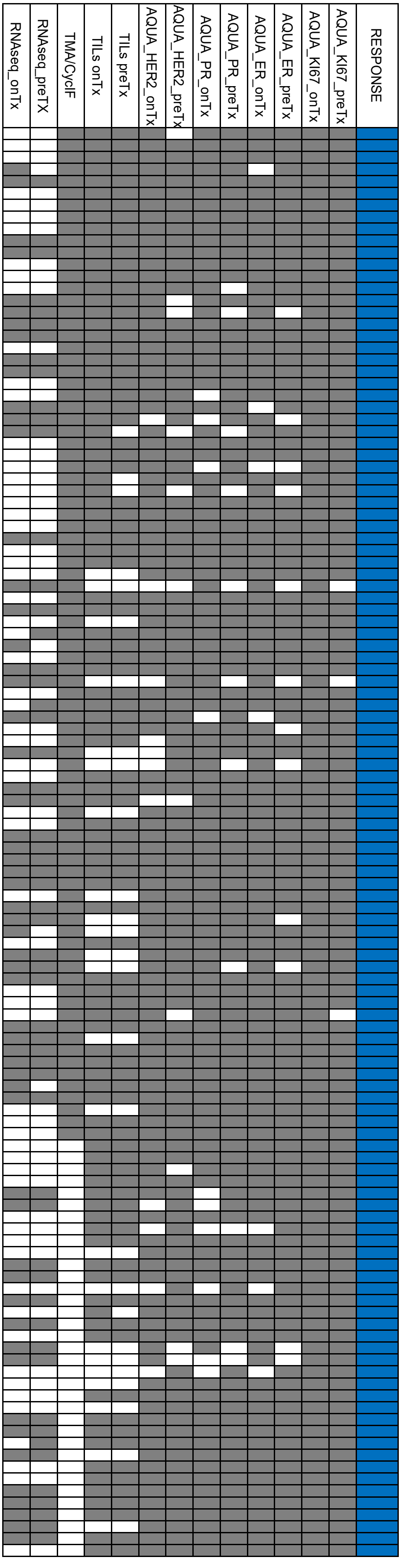

Supplemental Figure 11.
Tile plot of the patients samples used for the analysis
