# Supplementary material for: CD8+ T cells in the tumor microenvironment modulate the response to endocrine therapy in breast cancer
Source: J Clin Invest. 2025 Dec 9;136(3):e188458. doi: 10.1172/JCI188458 (PMC12867153; doi:10.1172/JCI188458)

Full unedited blot/gel for Figure 4F

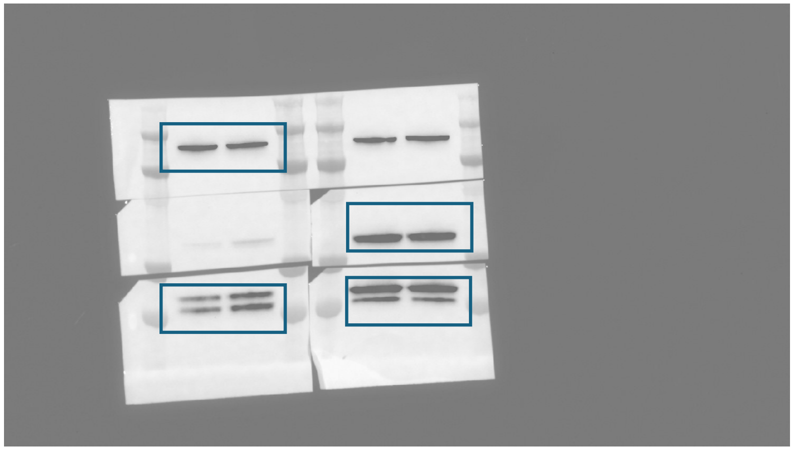

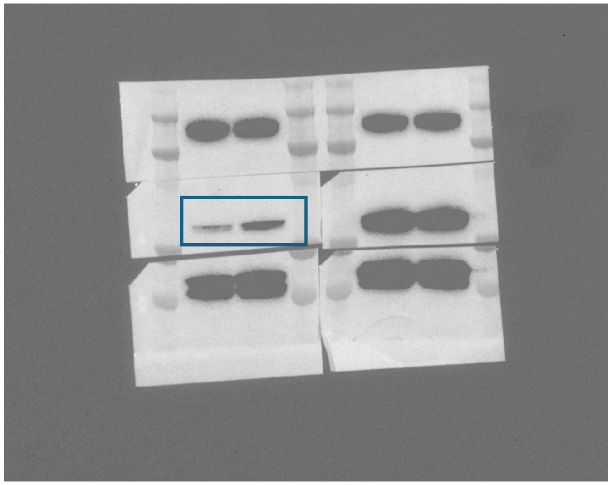

Full unedited blot/gel for Figure 4G

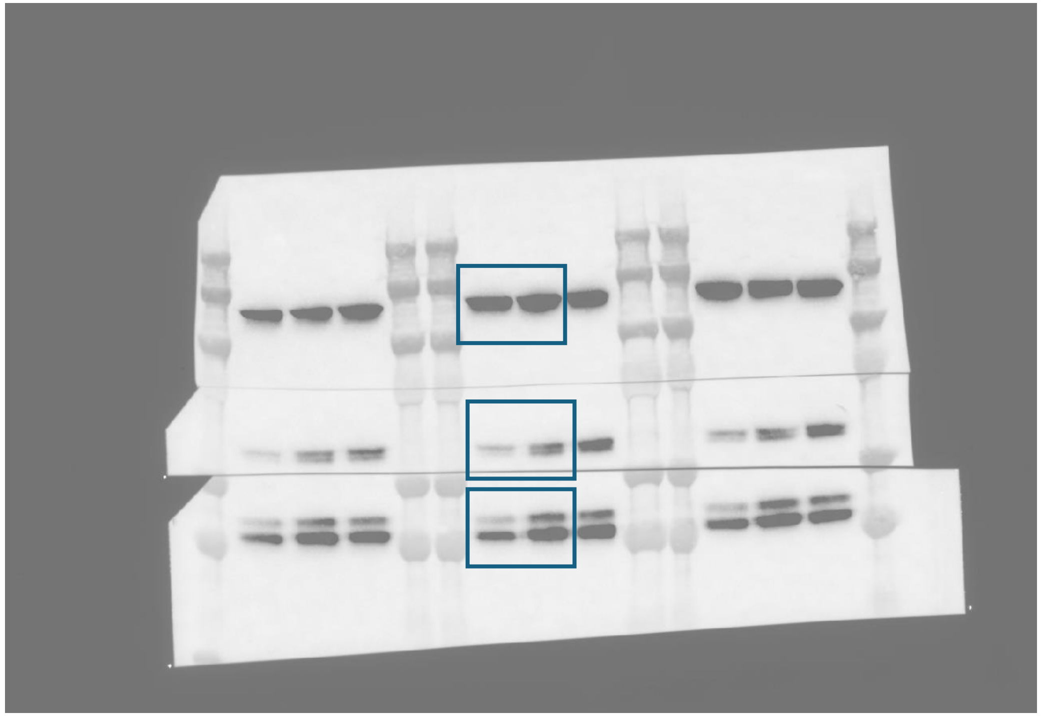

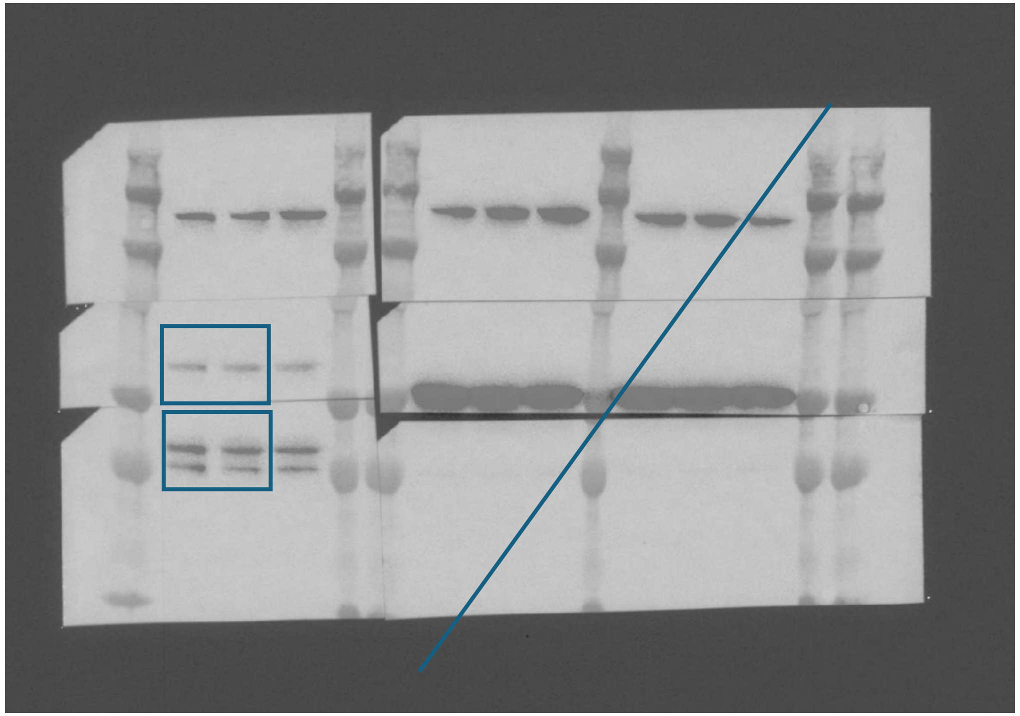

Full unedited blot/gel for Suppl. Figure 7F

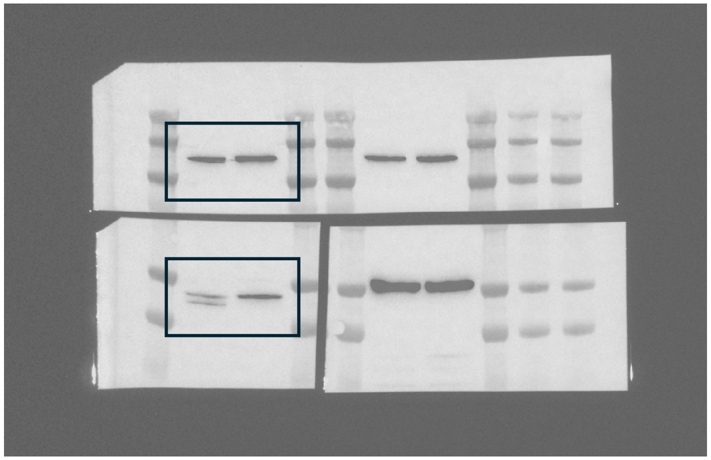

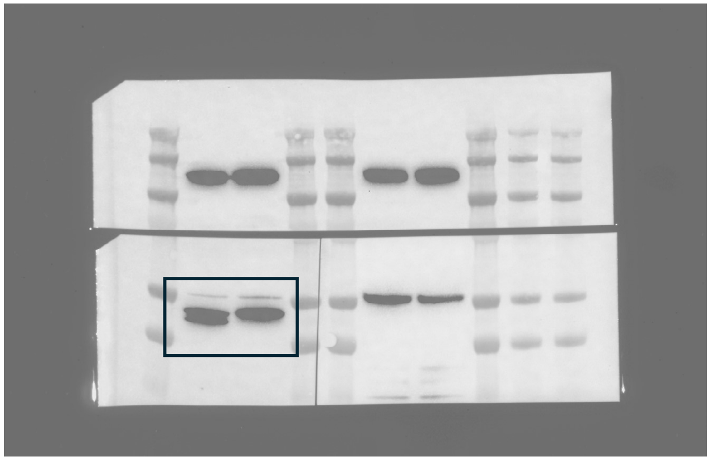

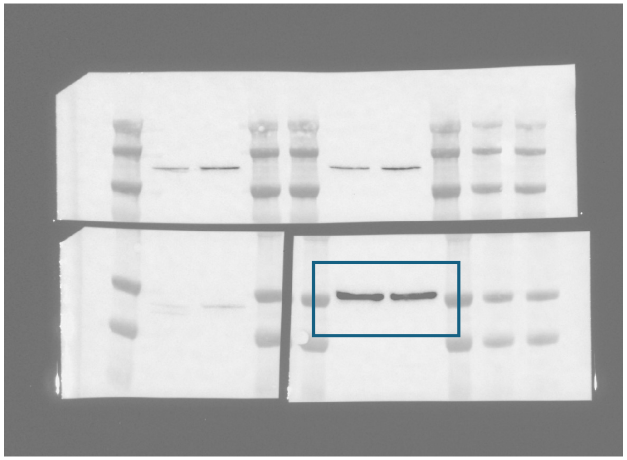

Full unedited blot/gel for Suppl. Figure 8D

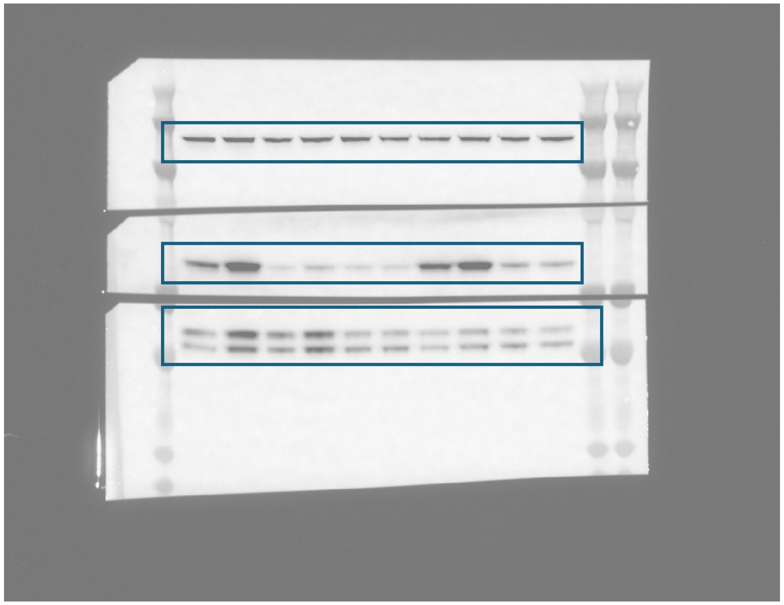

Supplement: Unedited blot and gel images [file jci-136-188458-s276.pdf]
